# The unintended negative consequences of artificial intelligence use for psychologists

**DOI:** 10.3389/fpsyg.2026.1729050

**Published:** 2026-05-18

**Authors:** Llewellyn E. van Zyl

**Affiliations:** 1 Psynalytics, Eindhoven, Netherlands; 2 Optentia Research Unit, North-West University, Vanderbijlpark, South Africa

**Keywords:** algorithmic dependence, artificial intelligence, ethical challenges, negative consequences of artificial intelligence, professional identity

## Abstract

The rapid integration of artificial intelligence (AI) into psychological practice is transforming how psychologists reason, document, and deliver care. While benefits such as improved diagnostic accuracy and administrative efficiency are widely recognized, the unintended consequences for psychologists themselves remain poorly understood. This commentary synthesizes recent empirical and conceptual literature to critically examine six domains through which sustained AI use may adversely affect psychologists’ professional functioning: (a) cognitive functioning and professional competence, (b) professional identity and meaning, (c) ethical reasoning and legal challenges, (d) interpersonal and social functioning, (e) data governance and vendor lock-in, and (f) personal wellbeing. The analysis draws on a narrative review of peer-reviewed sources from psychology, medicine, computer science, and organizational behavior, selected for their relevance to professional impacts of AI. Evidence suggests that cognitive offloading and automation bias may erode diagnostic reasoning and clinical judgment, moral deskilling and value misalignment can undermine ethical autonomy, and technostress, data dependence, and vendor lock-in pose growing threats to practitioner wellbeing and professional sovereignty. These dynamics indicate that AI operates not merely as a technical aid but as a transformative social and psychological actor that reshapes how psychologists think, decide, and relate to their work. The paper argues for the urgent development of evidence-based safeguards, practitioner education, and regulatory frameworks to ensure that AI enhances rather than erodes human competence and ethical reflection.

## Introduction

Artificial intelligence (AI) is rapidly infiltrating every area of psychological practice (Choudhury et al., 2025) and is fundamentally changing how mental health professionals work and how clients seek care (Prégent et al., 2025). Psychologists are increasingly using AI throughout the entire spectrum of psychological services ranging from administrative tasks (e.g., note taking, marketing) and data analysis to clinical report writing, psychodiagnostics, diagnostic decision support, and even treatment planning (Auf et al., 2025; Cross et al., 2024). At the same time, public interest in AI mental health applications has surged by 114% between 2023 and 2024, with the most common use of generative AI in 2025 being therapy and companionship (Zao-Sanders, 2025). Yet this unprecedented increase has occurred largely without systematic evaluation of its unintended consequences. While the discourse on AI use in mental health care has predominantly centred on clients, there is far less attention being paid to its effects on the psychologists themselves. The speed of AI adoption is dangerously outpacing our understanding of the risks it poses to the professional functioning and wellbeing of psychologists, perhaps because they are blinded by the genuine and substantial benefits these systems provide.

These benefits are substantial and well-documented in the literature. In psychological practice, AI tools can not only automate workflows and alleviate administrative burdens but are able to significantly enhance the psychologist’s clinical effectiveness (Barath et al., 2025). For example, machine learning algorithms demonstrate higher accuracy than humans in diagnosing psychological conditions ranging from depression and anxiety to schizophrenia, with accuracy rates between 83.33 and 99.79% (Dhariwal et al., 2024; Doki et al., 2021). AI systems can process vast amounts of clinical data, self-report measures, behavioral patterns, and treatment histories to identify subtle diagnostic patterns invisible to human observation (Dhariwal et al., 2024). These capabilities enable hyper-personalized treatment strategies tailored to unique psychological profiles, dynamic adjustments to therapeutic interventions, and early detection of psychopathology during prodromal stages when interventions are most effective (Curtiss and DiPietro, 2025).

Despite these benefits, emerging evidence from adjacent domains points to new forms of cognitive offloading, professional deskilling, ethical dissonance, and emotional strain arising from sustained AI use (Gerlich, 2025; Morrin et al., 2025). Recent research shows that nearly one in four people display symptoms of AI dependence, including compulsive checking behaviors and withdrawal distress when chatbot access is limited (Huang et al., 2024). Clinical cases of reality distortion after extended chatbot interaction are appearing in psychiatric clinics (Moore et al., 2025), and LLM chatbots consistently fail to implement basic safety standards, sometimes providing harmful information to users with suicidal ideation (Moore et al., 2025). More concerning, studies show that most psychologists have limited understanding of how these tools work, with their exposure coming primarily from mainstream media and commercial advertising rather than systematic training (Cross et al., 2024). Many psychologists admit to using AI tools without fully understanding their risks (Hipgrave et al., 2025).

As such, the purpose of this perspective paper is to critically examine the unintended negative consequences of AI integration and overuse of psychologists within psychological practice. Specifically, it explores how sustained engagement with AI systems may undermine psychologists’ cognitive competence, ethical reasoning, professional identity, interpersonal functioning, data governance responsibilities, and overall wellbeing. The central message is that without urgent recognition and mitigation of AI’s unintended harms, psychology risks undermining both its professional foundations and the very wellbeing it seeks to protect.

## Methodological approach

This paper represents a perspective commentary and narrative synthesis rather than a systematic review. The analysis draws on peer-reviewed empirical and conceptual literature from psychology, medicine, computer science, organizational behavior, and human-computer interaction. Sources were selected based on their relevance to the professional impacts of AI use, with particular attention to studies examining cognitive effects, professional identity, ethical challenges, and practitioner wellbeing. Given the recency of AI integration into psychological practice, the review incorporates findings from adjacent healthcare domains, particularly medicine, where AI adoption is more advanced and empirical evidence more developed. Where direct evidence from psychology is unavailable, extrapolations are clearly identified as such, and claims are appropriately hedged to reflect the current state of knowledge. This approach enables early engagement with an emerging phenomenon while acknowledging the limitations inherent in applying findings across professional contexts.

## How AI reshapes psychologists’ competence, identity, and practice

The integration of AI into psychological practice is not only improving professional efficacy and enhancing the quality of their care, but also has the potential to create a series of unintended pressures that cut to the core of professional functioning. Emerging evidence is starting to show that AI is reshaping how psychologists think, how they work, and how they relate to both their clients and colleagues—often in ways that undermine the very competencies the profession depends upon. The risks AI poses to psychologists are not just related to technical glitches and hallucinations of AI systems, but rather they span a series of six interconnected factors that may affect the quality of their care: (a) cognitive functioning and professional competence, (b) professional identity and the meaning of work, (c) ethical and legal challenges, (d) interpersonal- and social-functioning, (e) data governance and vendor dependencies and (f) impacts practitioner physical and mental wellbeing.

If these are left unchecked, it could create conditions where psychologists may become less skilled, less present, and less resilient, while simultaneously becoming more dependent on opaque systems that disempower them. Understanding these potential cascading consequences is essential if psychology is to preserve its distinctive expertise in an era where technological systems are increasingly starting to compete and occupy the space in which psychologists practice. In the sections below, we discuss each of the six interconnected ways AI might affect the inner and outer worlds of psychologists in more detail.

## Cognitive functioning and professional competence

AI systems are becoming deeply embedded in how psychologists think, reason, and make clinical decisions. While these tools can reduce cognitive burden and improve efficiency, their sustained use may fundamentally alter the cognitive architecture underlying clinical expertise. This section examines how AI engagement affects cognitive offloading, attention and memory processes, diagnostic skills, and metacognitive abilities (c.f. Table 1). Understanding how these processes unfold, and why psychologists remain largely unaware of their potential skill regression, is critical for preserving professional competence in an AI-augmented field.

**Table 1:** Cognitive functioning and professional competence: mechanisms and impacts on practice.

Issue/mechanism	Description	Impact on psychological practice
Cognitive offloading	Using AI to remember, organize, or write things so your own mind does less active thinking.	Skills develop more slowly, clinical reasoning weakens, and performance drops when the tool is not available.
Cognitive debt	The long-term cost of repeatedly letting AI think for you. Over time, core abilities fade from lack of use.	Harder to reason independently, recall key ideas, or make confident judgments without support.
Attention fragmentation	Dividing attention between the client, the screen, and AI prompts makes it hard to stay fully present.	Missed cues and emotional signals, weaker therapeutic connection, more mistakes during sessions.
Decline in working memory	When the tool holds the details, your brain practices holding and updating them less. The short-term mental workspace weakens and overloads more easily.	Harder to keep key facts in mind during sessions, slower to link themes, greater reliance on notes or AI to continue
Diagnostic deskilling	Relying on AI suggestions too early stops you from forming your own diagnostic hypotheses.	More diagnostic errors, less sensitivity to culture and nuance, overconfidence in AI-generated labels.
Deteriorating work quality	AI-generated notes look professional but often lack depth, context, and human meaning.	Records become longer but less useful, reflective thinking fades, and case formulations lose richness.
Automation bias	Over-trusting confident AI outputs while ignoring conflicting evidence or context.	Wrong recommendations followed, accountability weakens, and ethical or legal exposure grows.
Metacognitive deskilling	Depending on AI for validation erodes your ability to reflect and justify your own thinking.	Harder to explain or defend decisions, less adaptability in new situations, weaker supervision of trainees.

### Cognitive offloading and cognitive debt

*Cognitive offloading* represents one of the primary mechanisms through which AI use begins to affect a psychologist’s level of clinical competence. Cognitive offloading refers to the intentional delegation of mental processes such as recall, reasoning, and synthesis to external tools (Gerlich, 2025). While offloading can temporarily reduce one’s cognitive load/strain and increase perceptions of professional efficiency, it simultaneously prevents the effortful cognitive processing necessary to build and maintain expertise over time (Gerlich, 2025; Shukla et al., 2025). A 2025 mixed-methods study of 666 participants reported a strong negative association between frequent AI tool use and critical thinking which was significantly influenced by cognitive offloading behaviors (Gerlich, 2025). Although the study was conducted in a general population sample rather than with professional psychologists, this is one of the first large empirical demonstrations of the offloading–reasoning link in the AI era.

A landmark MIT Media Lab experiment studied how LLM-assisted writing affects the brain and future writing performance (Kosmyna et al., 2025). Researchers compared cognitive functioning of three groups in writing essays: one using LLM-assistance, one using search engines, and one writing from memory alone. Researchers found that participants who were instructed to use AI assistants to help produce academic level essays, started to show behaviors that were consistent with cognitive debt (Kosmyna et al., 2025). When the AI assistant was removed, there was a sharp decline in the writing performance of participants (Kosmyna et al., 2025). The researchers also looked at brain functioning during the experiment. The findings also revealed a systematic scaling of neural engagement based on the level of external cognitive support provided by the LLM-assistant (Kosmyna et al., 2025). The Brain-only group who did not use the LLM-assistant demonstrated the strongest neural network connectivity, while the Search Engine group showed intermediate engagement, and LLM-to-Brain group showed the weakest overall brain coupling (Kosmyna et al., 2025).

This study provides neurobiological evidence that cognitive offloading to AI systems does not merely create a temporary dependence on the system but rather produces lasting changes in one’s cognitive processing capacity. The systematic downregulation of neural networks in response to AI assistance suggests that the brain adapts to reduced cognitive demands by decreasing the strength and complexity of connections required for independent analytical work (Kosmyna et al., 2025). For psychologists, this implies that routine use of AI for clinical reasoning, case conceptualization, or treatment planning may not simply represent a reversible efficiency choice but could fundamentally reshape the neural architecture supporting their clinical expertise (Lee et al., 2025). This creates cognitive debt that accumulates over time and may prove difficult to remediate once its established (Lee et al., 2025).

The mechanism underlying this decline operates through what researchers term “cognitive debt” (i.e., the accumulated cost of relying on external systems for mental processing that would otherwise strengthen clinical competencies) (Gerlich, 2025). Cognitive debt refers to the accumulated cost of repeated cognitive offloading, where short-term ease is “financed” by the long-term erosion of professional skills and even memory (Kosmyna et al., 2025). According to Lee et al. (2025) users who frequently delegate cognitive tasks to AI tools, such as using algorithms for quick diagnostic suggestions or relying on recommendation systems for treatment decisions, exhibit weaker critical thinking skills and reduced capacity for independent analysis (Gerlich, 2025; Shukla et al., 2025).

### Attention fragmentation and working memory degradation

Prolonged AI use affects the attentional and memory processes underpinning professional competence (Gandhi et al., 2023; Naseer et al., 2025). Psychologists must simultaneously manage demands of monitoring AI outputs, managing digital interfaces, and verifying recommendations, all while maintaining therapeutic presence. These concurrent demands overload working memory capacity, leading to attentional fragmentation and mental fatigue. For psychologists, whose work depends on deep listening, empathic presence, and reflective awareness, this fragmentation undermines both clinical reasoning and therapeutic connection.

Cognitive load theory helps explain this process (c.f. Plass et al., 2010). Each new AI interface that a psychologist engages with introduces an additional cognitive demand. When these demands exceed one’s capacity for working memory capacity, psychologists start to shift from deliberate, reflective processing practices to more reactive, surface-level types of reasoning (Nizamani et al., 2024). This is done to reduce experiences of cognitive load, and to free up mental energy for other tasks (Plass et al., 2010). In therapy, this manifests as difficulties to sustain focus on clients’ narratives, slower recognition of their emotional cues, and a reduced ability to make accurate diagnosis. It also affects their ability to remember fine-grained details of earlier sessions which compromises the rapport they have established with the client (Plass et al., 2010).

Memory degradation represents a deeper consequence. Working memory behaves like a trained capacity that strengthens with deliberate practice and weakens when processes are routinely offloaded (Shanmugasundaram and Tamilarasu, 2023). Here three mechanisms converge: chronic digital load crowds the limited-capacity operational functions working memory depends on; AI encourages systematic offloading where focus shifts from remembering content to remembering where to find it (Storm et al., 2017); and extended AI use alters brain networks responsible for sustained attention and memory consolidation (Kosmyna et al., 2025; Small et al., 2020). This process makes psychologists progressively less capable of sustaining focused attention or forming robust memory traces without technological support (Small et al., 2020).

### Diagnostic deskilling

The *erosion of diagnostic skills* represents perhaps the second most concerning manifestation of AI-related competence degradation. Diagnostic deskilling occurs when reliance on automated decision support reduces the frequency and depth of clinicians’ own hypothesis generation and testing abilities (Budzyń et al., 2025). This phenomenon is well documented in healthcare research. According to Natali et al. (2025) documented widespread “deskilling” across medical specialties when professionals become overly reliant on automated diagnostic support systems.

A landmark study by Budzyń et al. (2025) examining endoscopists’ diagnostic performance provides the first real-world documentation of AI-induced deskilling in clinical practice. Researchers found that after extensive use of AI-assisted anomaly detection systems during colonoscopies, physicians’ unassisted adenoma detection rates dropped significantly from 28 to 22%. This implies a 6% decline in diagnostic accuracy when the AI assisted anomaly detection software was unavailable (Budzyń et al., 2025). Although from gastroenterology, it is the clearest real-world demonstration that continuous AI support can depress unaided diagnostic performance. This finding demonstrates that automated support may prevent the deliberate practice necessary to maintain expertise, even among experienced practitioners. If similar dynamics emerge in mental health, prolonged use of diagnostic or triage aids could reduce clinicians’ vigilance and pattern recognition when facing subtle or culturally atypical symptoms.

The mechanisms underlying diagnostic deskilling operate through multiple pathways. First, reduced practice of differential diagnosis generation occurs when psychologists increasingly rely on AI-generated diagnostic suggestions rather than systematically working through alternative explanations for the problems clients present with (Budzyń et al., 2025). Second, decreased confidence in clinical intuition develops as practitioners begin to doubt their own clinical judgment when it diverges from algorithmic recommendations (Duran, 2021; Natali et al., 2025). Third, an over-reliance on algorithmic decision trees may start to develop which is problematic because these systems may fail to capture the nuanced, contextual experiences of clients which are common in psychological practice where symptoms rarely conform to neat diagnostic categories (Klein, 2022).

The idea of developing an “inhibition to upskilling” may further compound the deskilling problem. AI systems not only erode existing competencies but also reduce opportunities for actual skill acquisition and development (Klein, 2022). For example, when intern psychologists begin to examine patients and learn about various pathologies, they may be inclined to diagnose, treat, or make decisions solely based on what AI systems recommend even without a thorough examination of the client (Klein, 2022). This pattern creates a dangerous trajectory where junior psychologists may never fully develop the foundational clinical skills that senior practitioners take for granted which may potentially create a generation of psychologists that are unable to function effectively without technological support.

### Deteriorating work quality

Paradoxically, when AI is used to help automate workflows and manage admin burdens, we may start to see a rapid *decline in the quality of work* produced by psychologists. We could start to see the quality of clinical reports decline despite using AI assistance systems that are explicitly designed to improve clinical record-keeping (Lee et al., 2024). While AI-generated clinical notes may be longer and more detailed, they often lack the clinical insight, therapeutic process observations, and contextual nuance that is usually characterized by high-quality psychological documentation (Lee et al., 2024). Over time this can thin the clinical narrative that underpins formulation and treatment planning. The convenience of AI-assisted documentation leads practitioners to become passive consumers of AI-generated content rather than active creators of meaningful clinical narratives (Lee et al., 2024). This means there might be a fundamental shift in how psychologists process and integrate clinical information (Asgari et al., 2025).

Further, reviews of AI scribes in healthcare settings show persistent and distinct failure modes ranging from active omissions of content, and misplaced attributions, to subtle hallucinations that require clinician correction (Lee et al., 2024). None of the evaluated systems were consistently error free (Lee et al., 2024). Related benchmarking work in digital medicine also shows small but nontrivial hallucination and omission rates in LLM-generated clinical summaries. For psychologists this mean if they shift from authorship of these notes to just light editing of AI-generated notes their reflective thinking may atrophy over time and their overall case memory may start to degrade. In other words, the record keeping and note taking might become more extensive but become clinically less meaningful.

### Automation bias and clinical decision-making

*Automation bias* poses another significant risk to *clinical decision-making* in psychological practice. This refers to the tendency to over-trust suggestions from automated systems, accepting concordant outputs and discounting any contradictory evidence (Goddard et al., 2014). Automation bias manifests through two distinct types of errors: (a) commission errors (i.e., where clinicians follow incorrect AI advice despite possessing contradictory clinical information) and (b) omission errors (i.e., where psychologists fail to act because AI did not prompt them to do so) (Goddard et al., 2014). Both types of errors imply that there is an inappropriate kind of transfer of responsibility from a human psychologists’ clinical judgment to algorithmic systems which undermines the level of critical thought and careful reasoning that underpins effective psychological practice. In practice this can lead to psychologists uncritically adopting the recommendations of AI systems and reduce their ability for independent verification (Romeo and Conti, 2025).

This is clearly demonstrated in the medical sciences. A randomized control study by Qazi et al. (2025) found that physicians showed significant automation bias in their diagnostic reasoning when they used a state-of-the-art LLM, even when the AI was wrong. This indicates that LLM assistance can systematically shape a practitioner’s clinical judgments (Qazi et al., 2025). Broader human–AI reviews in 2025 confirm that automation bias is more prevalent in situations that require time-pressured decision making and that it affects both highly experienced practitioners and novices (Romeo and Conti, 2025). Although older, the foundational systematic review of automation bias in medicine shows it recurs across tasks and different user groups which reinforces concerns about its persistence when using newer LLMs that are a lot more sophisticated than in the past (Goddard et al., 2012). In practice this means that the time pressure usually involved in outpatient settings, coupled with the apparent fluency and confidence in which LLMs presents their outputs, may create fertile ground for automation bias to occur. This in turn can dampen a psychologist’s natural discrepancy-seeking behaviors which could lead to the premature closure of complex cases - especially when the AI echoes psychologists’ initial impressions or assumptions.

The factors mediating automation bias might explain why psychologists are especially vulnerable to automation bias. User factors such as task-specific experiences, cognitive processing styles, trust in technology, and confidence in one’s own abilities all influence one’s susceptibility to automation bias (Goddard et al., 2014). Environmental factors like workload, task complexity, and time constraints that put pressure on one’s cognitive resources might further increase reliance on AI recommendations (Romeo and Conti, 2025). Interestingly, even experienced physicians showed to be vulnerable to automation bias, particularly when they are under high cognitive load or when AI systems present recommendations with high confidence levels (Romeo and Conti, 2025). This means that experience alone is not enough to protect one against automation bias and that active awareness and deliberate strategies are necessary to maintain an appropriate level of clinical scepticism.

### Metacognitive deskilling

Finally, when all these factors combine, it might lead to a decline in *metacognition*. Metacognition is a psychologist’s capacity to monitor, regulate and question their own thinking (Tankelevitch et al., 2024). Healthy metacognition includes an acute awareness of uncertainty, having the ability to justify choices, and being able to reflect and revise one’s thinking (Tankelevitch et al., 2024). If psychologists outsource both their work-related content and their confidence to AI systems, then their metacognitive monitoring will also tend to deteriorate over time (Lim, 2025; Tankelevitch et al., 2024). This kind of metacognitive deskilling creates a troubling scenario where psychologists become unable to articulate the reasoning behind their clinical decisions. This may potentially compromise their ability to justify the choices of their interventions, lead to an inability to adapt to novel situations, or being unable to teach clinical reasoning to intern psychologists. The loss of metacognitive awareness is not just a loss in a technical skill but rather a fundamental erosion of the reflective practice that characterizes what makes a psychologist an expert diagnosis and interventions.

## Professional identity and meaning

Beyond cognitive impacts, AI integration reshapes the foundational structures of professional identity, meaning, and ethical practice. This section examines how AI threatens professional identity, creates meaning crises, and generates novel ethical dilemmas (c.f. Table 2). Understanding how these professional identity threats may unfold is essential for preserving psychologist’s sense of purpose.

**Table 2:** Professional identity and meaning: identity threats and consequences for practice.

Issue/mechanism	Description	Impact on psychological practice
Identity threats and fear of replacement	When AI starts handling core psychological tasks, psychologists feel their expertise and independence are being replaced or devalued.	Increased anxiety and defensiveness, avoidance of AI tools, reduced confidence in judgment, and a shrinking sense of professional worth.
Role fragmentation and erosion of self-concept	Work becomes a series of small, tool-driven tasks rather than a cohesive craft. Psychologists start acting more like editors of AI outputs than clinicians.	Blurred professional boundaries, weaker reflective practice, declining engagement, and a loss of pride in the quality of one’s work.
AI-specific impostor syndrome	Success feels inauthentic because AI made the work easier. Comparing oneself to machine efficiency can erode confidence.	Growing self-doubt, over-reliance on AI for validation, hesitation to tackle complex cases, and reluctance to mentor others.
Meaning crisis at work	The more time spent managing systems and less on human connection, the harder it becomes to see the deeper purpose in the work.	Lower motivation, emotional fatigue, rising burnout, and reduced empathy and presence in sessions.
Intergenerational divide and cohesion	Younger psychologists trained with AI and older ones trained without it develop different habits and standards	Supervision conflicts, inconsistent quality of care, tension in teams, and weaker collaboration across experience levels.

### Professional identity threat and fears of replacement

When AI capabilities overlap with traditional psychological competencies, it creates profound *threats to one’s professional identity*. Identity threats occur when core tasks that define a profession are either taken over by other fields (e.g., HR practitioners using psychometric tests) or if these are automated or devalued (Van Zyl et al., 2016; Wagner and Schwind, 2025). This results in practitioners questioning what their unique value proposition is and fuels fears of becoming obsolete or being replaced (Wagner and Schwind, 2025).

Research on medical professionals reveals that identity threats operate through two mechanisms: challenges to professional expertise when AI demonstrates superior performance on tasks practitioners have mastered, and challenges to professional autonomy when AI constrains professional discretion (Jussupow et al., 2022). Medical students experience particularly strong identity threats and higher resistance compared to established practitioners (Jussupow et al., 2022). These threats are captured by “Artificial Intelligence Replacement Dysfunction” (AIRD), describing the existential and psychological distress professionals could experience when confronted with the threat of being replaced by AI, characterized by anxiety, insomnia, demoralisation, and identity confusion (McNamara et al., 2025). Wagner and Schwind (2025) found that German psychologists expressed fears about AI impacting their work and leading to professional obsolescence, with acceptance moderated by perceived usefulness but constrained by professional values. In Saudi Arabia, nearly half of psychologists surveyed feared that AI will alter their clinical role (Sharif et al., 2025).

These identity threats and fears of being replaced are compounded by a lack of awareness of these technologies as well as poor education about and formal training in AI (Arvai et al., 2025). When practitioners struggle to understand what AI is, what it can and cannot do, and what their role in its development/deployment is, it creates uncertainty about the role and relevance of human expertise in providing care (Arvai et al., 2025). This uncertainty regarding one’s utility may contribute to fears of professional replacement which is difficult to address through reassurance alone (Wagner and Schwind, 2025). This is because practitioners may lack the knowledge and skills needed to critically evaluate AI’s actual capabilities versus marketing claims (Arvai et al., 2025) as well as show an inability to articulate their unique value proposition in providing quality care (Wagner and Schwind, 2025).

This creates an existential dilemma: if the nuanced, individualized understanding that psychologists bring to each client can be replicated by an AI, and if a psychologist is not able to articulate what additional value they add to the process, what remains of their professional distinctiveness?

### Professional identity erosion and role fragmentation

*Professional identities* tend to erode through a gradual change in how psychologists understand and articulate their role and value (Van Zyl et al., 2016). These professional identities naturally change over time as new developments in a field emerge, new technologies are introduced, as the needs of clients/stakeholders changes and as new legislations are adopted (Van Zyl et al., 2016). However, professional identities start to erode when these developments start to fundamentally alter the very nature of their work by decomposing their professional activities into discrete and optimizable subtasks that can be automated, measured and optimized (Aquino et al., 2023). For example, in AI-enabled workflows, practitioners are increasingly feeling that they are reduced to “data entry clerks” or “AI supervisors” rather than being skilled clinicians (Aquino et al., 2023). This represents a fundamental shift in and erosion of their professional self-concept due to the significant fragmentation and automation of their ‘traditional’ professional roles (Aquino et al., 2023).

When professional *roles are fragmented* and decomposed into discrete, optimisable subtasks it could lead to cognitive dissonance as psychologists may struggle to integrate AI-assisted tasks with their broader therapeutic identity (Ackerhans et al., 2025; Beg and Verma, 2025). For example, if psychological assessment becomes nothing more than entering client information into a system, or when treatment planning means merely selecting from a list of AI-recommended interventions, then the integrated clinical reasoning that once characterized expert practice start to fracture into nothing more than a series of disconnected activities (Aquino et al., 2023). This fragmentation could lead to confusion about professional boundaries (and ethical accountability) as well as uncertainty about what core competencies a psychologist actually should have (Ackerhans et al., 2025).

The shift from active creators of content and providers of services to passive curator of AI-generated content perhaps represent one of the most fundamental ways AI is eroding the professional identities of psychologists. Traditionally, psychologists build their professional identity through both professional training and effortful work (e.g., grappling with complex diagnoses and tailoring interventions to the nuances of each client) (Jorgensen-Graupner and Van Zyl, 2019). This continued process of struggle and synthesis laid the foundation for their professional confidence and self-worth. When AI systems can generate insights, make case formulations, or suggest treatment strategies with apparent ease, then there is no need for the psychologist to go through this identity forming struggles. The results will be a profession wide level of uncertainty about whether psychologists should remain skilled clinicians or whether they have been reduced to intermediaries that oversees exchanges between clients and algorithms.

### AI-specific impostor syndrome

Another emergent phenomenon which threatens professional confidence is what we call *AI-specific impostor syndrome*. We define this as a psychological phenomenon where psychologists experience a persistent level of self-doubt and feelings of intellectual fraud that specifically relates to their achievements, insights, or professional outputs that involved AI assistance—despite objective evidence of their competence and value-added contributions. This sense of inauthenticity arises in two primary ways. First, psychologists may feel that their professional successes “do not count” because it is mediated or facilitated by AI. This undermines the link between effort, expertise, and achievement. Second, psychologists may begin to benchmark their own performance against AI systems themselves by comparing their own variability, fatigue, and uncertainty to the machine’s rapid, fluent, and seemingly tireless output. Together, these dynamics produce what we term AI-specific impostor syndrome, a novel form of professional self-doubt that’s rooted in both authenticity concerns and unfavourable comparisons with algorithmic performance.

This new form of imposter syndrome differs from the classical definition of the concept (c.f. Table 3 for a comparison). Classic impostor syndrome refers to feelings of inadequacy despite objective indicators of success or achievement (Clance and Imes, 1978). It stems from the irrational belief that professional success is undeserved or accidental and not attributable to one’s own skills, abilities or efforts (Clance and Imes, 1978). Here, individuals remain consciously aware that their successes or achievements required a substantial amount of sustained effort but still irrationally dismisses this effort by attribute success to external factors which ultimately leads to feelings of inadequacy or incompetence (Clance and Imes, 1978).

**Table 3:** Difference between classical and AI-specific imposter syndrome.

Aspect	Classic impostor syndrome	AI-specific impostor syndrome
Definition	Persistent self-doubt and feelings of fraudulence despite objective evidence of competence and achievement (Clance and Imes, 1978).	Persistent self-doubt and feelings of fraudulence specifically related to professional outputs facilitated by AI, despite clear evidence of competence and value-added contributions.
Core mechanism	Success is discounted because individuals irrationally attribute it to luck, timing, or external factors rather than their own abilities.	Success feels inauthentic because it required “too little effort” due to AI mediation, or because personal outputs are unfavourably compared to AI’s speed, fluency, and reliability.
Role of effort	Effort is acknowledged but devalued; achievements feel undeserved despite hard work.	Effort is minimized; achievements feel hollow precisely because they were too easily attained with AI assistance.
Comparison standard	Human peers or colleagues (e.g., “I am not as competent as others think I am”).	AI systems (e.g., “I am slower, less consistent, and less capable than the machine”).
Attribution pattern	Success attributed to luck, timing, or external circumstances rather than ability.	Success is primarily attributed to AI capability rather than human skill.
Ownership concerns	Doubts about whether recognition is deserved for genuine efforts.	Uncertainty about legitimate ownership of outputs when AI contributed significantly.
Competence assessment	“I’m not as capable as others think I am”	“I cannot tell which parts reflect my real competence versus AI augmentation.”
Intelligence framework	Intelligence validated through sustained struggle, effort, and mastery.	Distress over shift from effort-based to access-based intelligence
Fear focus	Fear of being “found out” as less competent than perceived	Fear that without AI support, one’s “true” incompetence would be revealed.
Psychological basis	Rooted in cognitive distortions and perfectionism; difficulty internalizing success.	Rooted in authenticity concerns (effort justification) and unfavourable upward comparisons with AI.
Professional identity	“I do not belong in this position”	“What constitutes my unique professional contribution?”
Validation source	Seeking validation through increased effort and perfectionism	Uncertainty about how to validate competence in AI-augmented context

AI-specific impostor syndrome, on the other hand, arises from the opposite. In classical impostor syndrome, individuals are aware of but undervalue or dismiss their efforts (Clance and Imes, 1978). In contrast, AI-specific impostor feelings arise precisely because success or accomplishments required too little effort because it was facilitated by AI. Achievements that once depended on deep clinical reasoning or creative problem-solving can now easily be produced with AI assistance which leads psychologists to question the authenticity of their own contributions. This dynamic reflects a level of cognitive dissonance where valued professional outcomes are achieved without the expected investment of effort, and this mismatch causes some level of discomfort about one’s own legitimacy (Rosenfeld et al., 1984). The phenomenon is further explained by the principle of effort justification, whereby people tend to value outcomes more and attach more meaning to achievements that required hard-work or effort (Rosenfeld et al., 1984). AI use therefore triggers this psychological mechanism by making achievements or successes feel more like “curation than generation” which challenges our ideas of what constitutes professional competence.

This phenomenon creates a paradoxical situation where both traditional indicators of a psychologist’s professional mastery (e.g., achievement) and doubt about professional competence coexist. In other words, psychologists may be able to produce high-quality work but at the same time can question whether they really have the skills and competence needed that the work they produced appears to demonstrate. In this way, professional achievement shifts from being grounded in effort and experience to a process of curating and refining machine-generated content. The result is a fundamental reconfiguration of how practitioners evaluate their competence and how they build professional self-worth.

The problem may be further exacerbated when psychologists begin to evaluate their performance against AI systems rather than human peers (Asbach et al., 2025). Research on AI users provides some evidence as to the prevalence of this phenomenon. Nearly half of AI users believe their AI assistant is (or will become) smarter than they are, while a significant percentage report concerns about dependence on AI for thinking and decision-making (Huang et al., 2024; Yigitcanlar et al., 2024). In the US, Rainie (2025) 52% of adults reported using LLMs and 49% believed the models they use are smarter than they are. This perception that can invite upward comparisons that depress self-efficacy (Huang et al., 2024; Yigitcanlar et al., 2024). A parallel YouGov (2025) poll found that 47% of Americans think AI is likely to become more intelligent than people which further reinforces the belief that machine competence will eclipse human skills over time. Although this data is not specific to psychologists, they provide some evidence that a comparison climate is starting to emerge which that professionals also inhabit.

There is also converging evidence that reliance on AI can grow over time and that overreliance on these systems carries cognitive and motivational costs that could fuel impostor-like symptoms of self-doubt. A longitudinal study found a growing trend toward psychological dependence on AI, with AI reliance rates rising from 17.14% at baseline to 24.19% as psychological reliance develops over time (Huang et al., 2024). Research also demonstrates that when employees feel outperformed by AI systems that they also experience negative emotions ranging from envy, and self-doubt to threats to self-esteem which all significantly undermines their willingness to collaborate with AI technology (Asbach et al., 2025). This creates an uncomfortable comparison dynamic where psychologists may start to benchmark themselves not against human colleagues (as in traditional impostor syndrome) but against AI machines that are potentially better, faster, smarter and more efficient than they are (McKee et al., 2023; Glickman and Zhang, 2024).

Take together, we believe that this phenomenon is more likely to manifest in professional environments that prioritizes speed, reliability, predictability, and net output quantity over quality and care. These are precisely the values that AI systems are designed to deliver (Glickman and Zhang, 2024). When clinical settings are increasingly starting to prioritize these kinds of metrics, psychologists may face increased pressure to adopt AI tools to help maintain their level of productivity while simultaneously starting to experience their slower, more deliberative human processes as being inadequate (Kline, 2022). This creates conditions where practitioners feel like impostors not because they lack competence but because their competence manifests through characteristically human processes that appear inferior when judged by machine-optimized criteria.

### The existential meaning crisis

The existential questioning about what one’s unique value is in one’s work results in what researchers term a “meaning crisis” (Seligman, 2012). In the context of AI, a meaning crisis occurs when one loses one’s sense of purpose when technology starts to mediate or even substitute parts of the psychological services that psychologists view as being intrinsically human. As AI systems demonstrate increasing capabilities in empathic responding, pattern recognition, and creative problem-solving, psychologists may struggle with fundamental questions about whether their presence offers irreplaceable value in the therapeutic process (Wagner and Schwind, 2025). At the individual level, practitioners who specifically decided to join the profession to provide human connection and healing may find themselves increasingly managing AI-mediated systems and interactions (Wagner and Schwind, 2025). This discrepancy between original professional aspirations and current technological reality leads to lower levels of meaningful work, ultimately affecting wellbeing (Van Zyl et al., 2010). Research on psychologists’ wellbeing shows that having meaning in and deriving meaning from work serves as both a critical protective factor against burnout and a main driver for wellbeing (Van Zyl et al., 2010). At the organizational level, if cultures prioritize efficiency and compliance with AI systems over relational depth and reflective practices, psychologists may feel pressured to compromise on what they consider the most meaningful aspects of their work (Kernes and Kinnier, 2008).

At the professional and disciplinary levels, the crisis manifests as collective uncertainty about what defines psychological expertise in an AI-augmented world. If machines can perform many technical functions of psychological practice, the profession must grapple with questions about the value it brings and what makes it distinct from AI-assisted care (Meadi et al., 2025). Professional societies have been slow to issue comprehensive guidance on AI, leaving members to navigate uncharted ethical terrain without clear collective voice. This creates a vacuum where commercial vendors, rather than professional bodies, implicitly set standards for psychological practice. The epistemological challenge is equally profound: as knowledge production becomes increasingly mediated by algorithms, psychology risks surrendering its authority in defining human experience to adjacent fields like computer science and data science (Van Zyl, 2026a,b). Research questions may shift toward those dictated by available datasets and computational power, away from questions with theoretical and clinical significance. At the societal level, as AI systems become more accessible and cost-effective, there is a growing cultural narrative that machines may serve as adequate substitutes for human-led professional care (De Freitas et al., 2024a,b). This risks reframing psychology as a set of commodifiable functions rather than a profession grounded in ethical responsibility, cultural sensitivity, and relational expertise.

The emerging meaning crisis is a cascading phenomenon where threats at each level amplify those at others, creating systemic vulnerability that threatens both practitioners and the profession itself. Individual psychologists struggling with existential questions find themselves working within organizations that structurally devalue meaningful aspects of their work, guided by professional societies that provide insufficient direction, operating within a discipline slowly losing its epistemological authority, and practicing in a society increasingly viewing their work as dispensable. This multilevel crisis highlights that AI’s threat to psychology extends beyond professional efficacy and cognitive deskilling as it strikes at its existential core: the sense of purpose, value, and meaning that sustains both individual practitioners and the collective professional enterprise.

### Intergenerational divide and professional cohesion

The *intergenerational divide* in AI adoption creates additional challenges to professional identity that threatens professional cohesion. Younger practitioners who trained with AI tools may never develop certain traditional competencies that senior clinicians consider foundational to psychological expertise (Aquino et al., 2023; Ta’an et al., 2025). Conversely, experienced psychologists may struggle to integrate new technologies into their established practices and may therefore see new technologies as threatening rather than something that can enhance their professional identity (Aquino et al., 2023; Ta’an et al., 2025). This technological divide risks fracturing the shared professional identity that has historically unified psychological practice across different career stages. Transferring knowledge and wisdom across generations becomes complicated when junior and senior practitioners operate with fundamentally different technological scaffoldings (Ide, 2025). Clinical supervision traditionally involves a senior psychologist modelling their clinical reasoning processes by demonstrating how they think through complex cases (Jorgensen-Graupner and Van Zyl, 2019). When supervisors and intern psychologists use fundamentally different tools or when one party relies heavily on AI-generated recommendations that the other cannot understand or evaluate, the relationship is poised to break down (Ide, 2025).

This generational tension leads to debates about professional standards of care, scope of practice and credentialing. Should licensing examinations assess AI-augmented clinical competence or traditional unaided abilities? Should continuing education requirements include AI literacy? How should professional ethics codes address AI use? The lack of consensus on these questions creates uncertainty that exacerbates the identity challenges discussed earlier. Different generations may hold incompatible visions of what psychological practice is or should become, which has the potential to further fragment professional identity and weaken collective professional advocacy. Professions derive strength from shared identity, values, and practices that unify members across contexts (Goto, 2021). When AI adoption creates fundamental disagreements about what constitutes competent practice, what skills matter most, and what role technology should play, it undermines the shared professional foundation. The result may be a fragmented field where practitioners no longer share a common professional language, values, or identity, weakening the profession’s ability to advocate for its members, maintain standards, or adapt collectively to future challenges.

## Ethical and legal challenges

The cognitive and identity challenges psychologists face are compounded by emergent ethical dilemmas that traditional professional frameworks struggle to address. As AI systems become more deeply embedded in practice, psychologists face cascading ethical challenges ranging from individual moral distress to systemic questions about professional responsibility, informed consent, and legal liability (see Table 4).

**Table 4:** Ethical and legal challenges: dilemmas, accountability, and informed consent.

Issue/mechanism	Description	Impact on psychological practice
AI-mediated moral injury and ethical deskilling	Psychologists may feel pressured to follow AI recommendations that conflict with their own judgment or client needs, leading to ethical strain and loss of confidence in moral reasoning.	Moral distress, erosion of integrity, emotional exhaustion, and growing dependence on AI to decide what is “right.”
Diffusion of professional liability	When AI influences decisions, it becomes unclear who is responsible for outcomes: Is it the psychologist, the organization, or the developer?	Increased legal exposure, defensive or inconsistent use of AI, and constant anxiety about accountability.
Accountability and ownership gaps	When AI produces recommendations, clinicians may feel less personally responsible for their effects. Over time, vigilance fades.	Weaker documentation, less ethical reflection, reduced learning from mistakes, and blurred professional responsibility.
Hidden AI use and loss of transparency	Using AI tools in therapy or documentation without disclosing it to clients violates honesty and informed consent principles.	Trust breakdowns, damaged therapeutic relationships, possible ethics complaints, and privacy breaches.
Black box opacity of AI systems	Many AI systems are impossible to fully explain, making it hard for both psychologists and clients to understand how decisions are made.	Incomplete or meaningless consent, inability to justify recommendations, and higher risk of biased or unsafe advice.
Algorithmic value imposition	AI systems carry the values and priorities of their developers, which may conflict with clients’ cultures, values, or therapeutic goals.	Reduced cultural sensitivity, pressure toward standardized care, loss of therapist autonomy, and ethical misalignment with clients.

### AI-mediated moral injury and ethical deskilling

*AI-mediated moral injury* is an emerging ethical threat which psychologists need to navigate these kinds of moral injuries occur when psychologists are forced or pressured to act in ways that conflict with their own core moral values. In AI-augmented practices, psychologists might feel compelled to follow algorithmic recommendations which may be counter-intuitive, contrary to their professional judgment or against the best interests of their clients due to institutional pressures to adopt AI, governmental policies or personal productivity demands (Mewborn et al., 2023). While the term is new in mental health, evidence from healthcare supports these analogous phenomena. Two recent literature reviews demonstrate increasing instances where practitioners experience moral distress that directly results from AI use (Mennella et al., 2024; Mewborn et al., 2023). This is a form of psychological distress or suffering that results from situations where one knows the ethically appropriate action to take but cannot act on it due to institutional or systemic factors related to AI use and deployment (Mennella et al., 2024). This AI-induced moral distress is particularly salient when organizations press for AI recommendations that conflict with clinical judgment (Mennella et al., 2024), especially when practitioners believe alternative approaches would better serve their clients (Mewborn et al., 2023; Pham, 2025). These pressures usually originate from organizations trying to implement AI for cost-efficiency (Pham, 2025), creating moral conflicts with practitioners’ fundamental imperatives of non-maleficence, beneficence, justice, and autonomy (Mewborn et al., 2023).

This AI-mediated moral injury extends to systematic challenges affecting professional autonomy and ethical reasoning (Mittelstadt, 2019). Moral deskilling refers to the gradual atrophy of a psychologist’s capacity for ethical reasoning which stems from repeated delegation of moral judgments to AI systems (Cohen et al., 2024). As AI decision engines embed more normative trade-offs, psychologists may increasingly defer their judgment to these systems, weakening their ability to handle novel or complex moral dilemmas over time. When AI platforms embed ethical considerations within recommendation algorithms, psychologists may progressively lose their ability to engage in the nuanced moral reasoning required for complex clinical situations (Mittelstadt, 2019). Ethical dilemmas in psychology often involve weighing competing values, cultural considerations, and contextual factors that AI systems cannot understand or address (Weiner et al., 2025). The convenience and apparent authority of AI-generated ethical guidance could create a pattern where psychologists stop engaging in the effortful moral deliberation that develops and maintains ethical expertise (Weiner et al., 2025). Over time, this could produce practitioners who can recognize ethical problems but lack the practiced capacity to independently reason them through.

### The diffusion of professional liability

The *diffusion of liability* in AI-assisted practice creates complex legal scenarios that current frameworks struggle to address. As AI suggestions are increasingly entering clinical workflows, the responsibility for their outcomes becomes harder to assign. Is the clinician liable for following AI, rejecting AI, or simply supervising AI output?

So, when adverse outcomes occur following an AI-influenced clinical decision, determining who is responsible becomes increasingly problematic as liability is potential being distributed across practitioners, AI developers, healthcare institutions, and the technology vendors (Maliha et al., 2021). Under current United States malpractice law, for example, liability rests on the “reasonable physician under similar circumstances” standard (Lindor et al., 2016). Here, courts judge the psychologist’s conduct regardless of whether AI was used or not, and there is currently no legislation that dictates responsibility with AI systems (even when their recommendations directly influence patient care) (Chew et al., 2025). This creates what legal scholars term the “liability sink” problem, where psychologists risk unfairly absorbing legal liability for errors and adverse outcomes over which they have limited control (Lindor et al., 2016; Terranova et al., 2024). However, the APA (2025) guidelines on ethical and responsible AI use clearly dictates that the psychologist remains ultimately responsible for the decisions about the patient.

*Legislation around liability with AI* use is in flux, and for now psychologists are operating in a deeply uncertain environment (Cestonaro et al., 2023). Malpractice claims involving AI are still relatively rare, but recent cases in the US are already highlighting how difficult it is for courts to determine responsibility when harm occurs (Cestonaro et al., 2023). Unlike medical practitioners, psychologists face relatively novel liability risks which range from failures to adequately supervise AI recommendations, the inappropriate reliance on algorithmic assessments without independent verification, and the inadequate disclosure of when AI was involved in treatment, to the inability to explain the reasoning behind AI-influenced clinical decisions (Terranova et al., 2024). It becomes even more difficult when trying to determine who is at fault, because it needs to be established if a psychologist deviated from “standard care” (Terranova et al., 2024). However, this becomes murky when the “standard” itself involves AI, and the AI is also constantly evolving and changing. This creates a kind of circular reasoning where psychologists may be liable for both inappropriately using AI and for failing to use it when others in their field do so (Price et al., 2024; Terranova et al., 2024). This legal ambiguity creates a problematic environment where accountability is not just structurally- but psychologically diffused (a dynamic we explore later in this section).

Beyond these legal and regulatory challenges lies an additional complication where *insurance and regulatory frameworks have also not kept pace with the risks associated with AI*. Many professional liability policies either completely exclude AI-related claims or use vague language that leaves psychologists uncertain whether they are covered or not (Rochester, 2025). The absence of clear regulatory guidance also makes the problem worse as there is no consensus on appropriate use standards. This uncertain liability landscape results in a professional environment where psychologists may feel obliged to use AI to remain competitive despite the fact that its legal and insurance protections are underdeveloped. This creates a misalignment between clinical innovation and regulatory safeguards.

### Professional accountability and psychological ownership of decisions

Yet beyond these legal and regulatory challenges lies an even more concerning problem. When recommendations are generated by AI, psychologists may f*eel less psychological ownership over its outcomes*. This creates a subtle moral accountability gap that further exacerbates the legal ambiguity (Königs, 2022). Research on AI-assisted decision-making demonstrates that individuals feel less responsible for the consequences of their decisions when these are AI-generated rather than self-authored (even when they technically made the final choice themselves) (Zeiser, 2024). Zeiser (2024) describe this as an “attributability gap.” This refers to a state in which a psychologist becomes uncertain about whom the value judgments embedded in, and the consequences of AI-assisted decisions should be attributed to (Zeiser, 2024). When AI systems are used to support decisions, they can function as “moral buffers” where psychologists are able to conveniently relinquish their psychological responsibility over the outcomes of their decisions (even if they still remain legally accountable for them) (de Santoni Sio and Mecacci, 2021). In other words, practitioners may unconsciously view the system as sharing or even totally absorbing the responsibility for outcomes which reduces their own feelings of accountability for the potential harm it may cause. This creates a kind of cognitive dissonance where psychologists remain legally responsible for the outcomes but at the same time experiencing diminished level of personal or moral responsibility because the decision felt driven by an AI system and not their own clinical judgment (Zeiser, 2024).

The potential reduction in psychological ownership is particularly concerning because it *weakens the internal sense of professional accountability* that ethical psychological practice fundamentally depends upon (Olckers et al., 2017). Psychological ownership theory establishes that individuals who have a higher level of involvement in processes or decisions tend to develop stronger feelings of responsibility for their outcomes (Olckers et al., 2017). When AI systems generate recommendations, psychologists’ direct involvement in the cognitive work associated with the decision-making process decreases. This leads to reduced levels of ownership of and accountability for both the decision and the potential outcomes (Chan C., 2025; Chan E., 2025). This creates practitioners who may view themselves as implementers of algorithmic directives rather than autonomous moral agents making consequential decisions about the welfare of their clients (Olckers et al., 2017). The combination of diminished psychological ownership, ambiguous legal liability, and absent regulatory guardrails creates a dangerous misalignment where psychologists may simultaneously feel less personally accountable for AI-influenced decisions while remaining fully legally responsible for their consequences (Wachter et al., 2017).

Over time, this dynamic could erode clinicians’ vigilance, weaken reflective ethical reasoning, and normalize a culture of diffused accountability where adverse outcomes are easier to dismiss as the fault of “the system.” The risk is not just legal exposure but the gradual deskilling of professional judgment itself, as psychologist may lose the habit of seeing themselves as the sole moral agents in their therapeutic decisions. This disconnection between the felt accountability and assigned responsibility threatens the ethical foundations of the profession.

### Hidden AI use and loss of therapeutic transparency

One of the more immediate ethical threats that psychologists might unknowingly engage in is the covert or hidden use of AI in psychological practice. *Hidden AI* use occurs when psychologists employ AI tools without disclosing that fact to clients (El Ali et al., 2024). These tools do not just relate to LLMs, Chatbots, therapeutic decision support tools, but also administrative and seemingly innocuous tools such as automated note-takers, Grammarly-style editors, or general-purpose large language models like ChatGPT. Yet even these “harmless” applications raise serious ethical and legal concerns, since most transfer sensitive clinical data to offsite servers that do not necessarily comply with HIPAA standards in the United States or with the stringent risk-management obligations mandated under the EU AI Act (Van Leeuwen et al., 2025).

Hidden AI use is far from rare. Meadi et al. (2025) infer that many mental health professionals use AI tools without explicitly informing clients and this could potentially not only violate professional ethics and legal standards. Even when AI is disclosed, most practitioners are under the impression that administrative AI applications like those mentioned above fall outside the scope of informed consent (Tavory, 2024). However, research demonstrates that non-disclosure has profound consequences for the therapeutic relationship. When clients discover undisclosed AI use (sometimes accidentally, such as observing therapists inputting session content into ChatGPT in real-time) it ruptures the bond with the therapist and could lead to feelings of betrayal, decreased trust, and reduced confidence in their provider’s competence (Van Zyl, 2026a). These kinds of ruptures matter because the therapeutic alliance is consistently one of the strongest predictors of successful treatment outcomes (Howgego et al., 2003), and its erosion may undermine the effectiveness of the client’s treatment regardless of the actual accuracy or utility of the AI system.

However, understandable, hidden AI use is not benign. It represents a fundamental breach of therapeutic transparency and informed consent, which deprives clients of their right to make meaningful choices about their care (Tavory, 2024). Ethical frameworks across professional psychology emphasize that client autonomy, honesty, and informed consent are non-negotiable principles of ethical and responsible practice. When psychologists integrate AI without disclosure, they not only compromise these ethical principles, but also substituting institutional or personal convenience for client agency. Over time, the normalization of hidden AI use risks embedding systemic deception into the profession which negatively affects public trust in psychologists and creates conditions in which technological mediation quietly reshapes therapy in ways clients neither understand nor endorse.

### The black box opacity of AI systems

The opacity of many AI systems creates new ethical and legal challenges that directly impact psychological practice. Because these models often function as “*black boxes*,” even the designers of these systems cannot reliably explain how inputs become outputs, such as why a given treatment recommendation was generated (McDermid et al., 2021). This poses significant risks related to transparency and accountability of decisions. When one cannot explain the reasoning behind a decision, it creates a scenario where meaningful informed consent is practically impossible (McDermid et al., 2021). Current legal frameworks require that clients receive sufficient information to make autonomous decisions about their care (Terranova et al., 2024), but how can patients provide informed consent when neither they nor their therapists understand how AI systems arrived at particular recommendations?

This opacity not only affects the explainability of decisions but also impedes the psychologist’s ability to detect errors, potential biases or inappropriate recommendations because these are based on incomprehensible algorithmic processes (Nebeker, 2024). Research demonstrates that AI systems can perpetuate and amplify biases present in training data, producing discriminatory outcomes that negatively affect marginalized populations (Obermeyer et al., 2019). When these systems operate as black boxes, neither psychologists nor clients can detect if AI recommendations reflect underlying biases rather than sound clinical reasoning (McDermid et al., 2021). The learned intermediary doctrine, which positions psychologists as intermediaries who can evaluate and explain treatments to clients, breaks down when the intermediary cannot understand or explain what they are intermediating (Price et al., 2024).

Regulatory compliance and practitioner legal obligations create further tensions. In many jurisdictions, the doctrine of informed consent requires psychologists to disclose how a proposed treatment strategy or diagnosis process will work, sharing risks, benefits, and uncertainties in a clear and understandable manner (Terranova et al., 2024). Courts examining black-box assisted medical decisions argue that practitioners who rely on opaque AI systems risk compromising respectful care if they cannot explain those decisions to patients (Cohen et al., 2024). The rapidly evolving nature of AI technologies vastly outpaces legislative and professional guidance (Terranova et al., 2024), and fragmentation of regulatory frameworks across jurisdictions creates confusion, especially when tools developed in one country are used in another. Some jurisdictions prohibit AI use in direct treatment decisions or client communications unless under oversight of a licensed professional (Tavory, 2024), while other jurisdictions provide no guidelines, leaving practitioners to navigate complex legal and ethical terrain without clear standards (Tavory, 2024). Professional bodies like the APA (2025) and the EFPA are struggling to provide definitive guidance due to the pace of technological change, meaning individual practitioners make consequential ethical decisions about client care without the benefit of professional consensus or peer support (Weiner et al., 2025). This combination creates an untenable situation: psychologists face pressure to adopt AI tools while simultaneously lacking clear guidance on appropriate use, adequate liability protection, or reliable methods for obtaining meaningful informed consent.

### Algorithmic value imposition: norms without consent

Even when AI systems are technically accurate or clinically useful, they embed values that are rarely disclosed, such as implicit priorities, trade-offs between efficiency and accuracy, and normative commitments. This results in what we call “algorithmic value imposition,” referring to the way AI systems, by virtue of their design, training objectives, and developer choices, enforce a particular set of assumptions about what good care means regardless of the client’s context or a psychologist’s views (Holm, 2024).

The first mechanism through which these values enter AI systems is through the specification of objective functions, loss functions, and performance metrics. Developers design systems with specific measurement priorities that embed normative trade-offs, dictating what counts as error, which outcomes are worth optimizing and how to balance accuracy with efficiency (Rajkomar et al., 2019). These are value-laden normative judgments dressed in technical language. No algorithm can be value neutral as all systems reflect the choices of their creators in weighting outcomes, prioritizing certain patient populations, or tolerating particular error types (Holm, 2024; Rajkomar et al., 2019). Once instantiated in model architecture, these normative priorities guide AI recommendations in subtle ways, steering clinicians toward particular treatment pathways as though they were clinical truths rather than value-laden design choices (Holm, 2024).

A second pathway involves value drift through systematic unintentional reinforcement feedback. When user behavior, psychologists’ treatment choices, or institutional incentives are fed back into AI retraining processes, the system naturally gravitates toward treating frequent patterns as normative (Swathi and Challa, 2023). Systems learn to suppress outlier approaches over time not because they are clinically unsound but because they deviate from efficiency norms or the most frequently occurring practice choice (Rajkomar et al., 2019). Because humans tend to trust AI outputs through automation bias, these hidden norms embed into daily practice while systematically deteriorating therapeutic pluralism.

Algorithmic value imposition threatens therapeutic pluralism by homogenizing practice around the AI’s embedded norms rather than psychologist reasoning or client values. AI systems trained on predominantly CBT-based datasets risk marginalizing other approaches such as psychodynamic, narrative, humanistic, or culturally adapted models which operate through mechanisms that algorithms cannot objectively capture (Straw and Callison-Burch, 2020). It may undermine cultural competence by encoding Western values like independence or assertiveness into systems as universal norms, misaligning care for clients from collectivistic cultures or relational traditions (Sedlakova and Trachsel, 2023).

When AI systems are trained on data predominantly from WEIRD (Western, Educated, Industrialized, Rich, Democratic) contexts, they may not recognize that concepts like independence, happiness, or emotional expression carry very different cultural meanings for those from non-WEIRD countries (Van Zyl and Rothmann, 2019). It also weakens self-determination, as AI recommendations implicitly substitute developer priorities for client goals and professional expertise. A client seeking meaning or spiritual growth may be steered toward standardized, symptom-focused interventions because the algorithm’s objective function fails to see these as legitimate therapeutic aims.

Over-reliance on AI tools can subtly pressure psychologists to align their views with algorithmic outputs even when these conflict with their professional judgment or understanding of a client’s values, risking deskilling in negotiating collaborative goals and weakening the ethical foundation of practice. When AI systems present recommendations as purely technical decisions, they obscure the fundamental value trade-offs involved in treatment decisions, potentially eroding psychologists’ reflective ethical reasoning abilities. Finally, because AI systems present recommendations in a convincing and authoritative manner, they could amplify existing power dynamics in therapeutic relationships. When a psychologist presents a strategy as “what the AI recommends,” clients may feel unable to advocate for alternatives or question recommendations (Mittelstadt, 2019), creating a form of covert paternalism where developer priorities silently shape treatment.

## Interpersonal and social functioning

Being an effective psychologist depends not only on technical competence and ethical clarity but also on the quality of relationships with clients, peers, and other stakeholders. Therapeutic presence, empathic attunement, and the transmission of tacit knowledge form the interpersonal bedrock of the profession. AI integration risks fragmenting attention, disrupting empathic communication, altering collegial exchange, and obscuring how clinical skills are cultivated (see Table 5).

**Table 5:** Interpersonal and social functioning: effects on presence, empathy, and collegial exchange.

Issue/mechanism	Description	Impact on psychological practice
Diminished therapeutic presence	Splitting attention between the client, screen, and AI tools makes it harder to stay emotionally and cognitively present in the moment.	Weaker therapeutic alliance, missed emotional cues, reduced empathy, and lower session quality.
Weakened nonverbal attunement	Watching interfaces instead of the client disrupts eye contact, timing, and body language synchrony.	Clients feel less seen and understood, leading to reduced trust and engagement.
Reliance on tools over peers	Turning to AI for advice instead of colleagues reduces shared reflection and professional dialogue.	Fewer diverse perspectives, more blind spots, weaker support networks, and greater risk of burnout.
Loss of tacit knowledge exchange	Standardized AI outputs strip away the subtle reasoning and contextual details normally shared between peers.	Diminished clinical intuition, less opportunity to learn from others, and a gradual erosion of professional wisdom.
Supervision masked by AI assistance	AI-generated reports and formulations can make trainees look more competent than they are.	Supervisors struggle to gauge real ability, independent reasoning weakens, and professional growth slows.
Uneven training and AI literacy	Programs differ on when and how trainees use AI, and there’s little agreement on what “AI competence” means.	Inconsistent professional standards, confusion about competence, and friction between generations of psychologists.

### The erosion of therapeutic presence

*Therapeutic presence* refers to a psychologist’s capacity to be fully engaged, aware, and responsive in the therapeutic moment at the physical, emotional, cognitive and spiritual level (Geller and Greenberg, 2012). Presence requires a mode of immediate perception that exists prior to any conceptual thought. Maintaining therapeutic presence is a foundational psychological skill and has long been linked to better client outcomes in psychotherapy (Geller and Greenberg, 2012). According to Baier et al. (2020) therapeutic presence helps build the therapeutic alliance, deepens the connection to the client, improves the extent clients self-disclose, and reduces the time it takes for clients to recover.

Presence requires undivided attention and becomes fragile when attention is divided (Baier et al., 2020). Managing AI prompts, fidgeting with note-takers, or typing client inputs during sessions competes with limited attentional resources. Even subtle task-switching can negatively affect the interpersonal synchrony on which presence depends (Norcross and Lambert, 2018). Clients are sensitive to these shifts, and studies indicate that patients notice and negatively interpret even brief lapses in practitioner attentiveness (Norwood et al., 2018). Studies of teletherapy show that technical demands create a “filtering effect,” reducing socioemotional cues that can impair the therapeutic relationship (Seuling et al., 2024).

The erosion of presence also carries psychologist-specific consequences. Presence is not only what the client perceives but its also how psychologists experience themselves in the room. Being fully present in the moment affects the psychologist’s confidence, creates a sense of flow, and reduced the effect that their emotional labour has on their mental health (Geller and Greenberg, 2012). However, when attention is persistently fragmented by AI systems or by the over-reliance on algorithmic recommendations, psychologists may start to experience their own work as being mechanical, or even meaningless. Finally, presence is also central to the supervision and training of future psychologists. Intern psychologists learn presence through watching how their supervisors inhabit the room, tolerate ambiguity, and stay attuned. If supervisors themselves are splitting focus between client and AI, the tacit modelling of presence risks being diluted.

### Weakened nonverbal communication and empathic attunement

The importance of nonverbal communication in conveying empathy and establishing therapeutic alliance has been well-established in psychotherapy research (Geller and Greenberg, 2012). Showing empathy in psychotherapy is the result of a dynamic interplay between facial expressions, body posture, vocal tones, and timing that conveys emotional attunement to the client (Elliott et al., 2018). Decades of research demonstrate that between 60–70% of interpersonal meaning is communicated through nonverbal channels (Mehrabian and Ferris, 1967). In therapy, nonverbal synchronicity tends to predict a stronger therapeutic alliance and leads to better treatment outcomes (Geller and Greenberg, 2012). These behaviors allow clients to feel understood at a level beyond words and the tacit skillset to demonstrate such is a fundamental element of being an effective psychologist (Jorgensen-Graupner and Van Zyl, 2019).

Research on technology-mediated therapy sessions already highlight how fragile nonverbal attunement can be (Simpson and Reid, 2014). In emotionally charged videoconferencing therapy sessions, research shows that clients’ perceptions of reduced eye contact, facial expressiveness, and gesture synchrony weakens the relational bond with their psychologist (Norwood et al., 2018; Simpson and Reid, 2014). Eye-tracking research in medical and counselling contexts shows that when practitioners direct their gaze more often to a screen than to the client, then clients perceive them as being far less empathic and see them as less trustworthy (Almansour et al., 2023). Even small delays in audiovisual transmission can disrupt the micro-timing of empathic responses that are critical to a client’s felt sense of connection (Geller and Greenberg, 2012). These findings demonstrate that any technology that diverts attention from the client can diminish empathic communication, even when verbal exchanges remain intact.

So we believe that AI systems used during therapy could amplify these vulnerabilities by introducing new competing demands on attention during the therapeutic encounter. When a psychologist consults real-time AI outputs, their gaze and facial orientation are pulled away from the client. These small attentional shifts translate into missed micro-expressions, fewer nods, or delayed empathic responses. Clients, who are acutely sensitive to perceived therapist distraction, may experience this as disconnection or lack of care. For the psychologist, repeated reliance on AI-generated recommendations (e.g., suggested responses) during a session could lead to a gradual weakening of their ability to generate spontaneous empathic behaviors. Over time, this could reshape not only session dynamics but also practitioners’ confidence in their own empathic abilities, which may undermine both professional identity and competence.

### Professional relationships and collegial consultation

Beyond the immediate therapeutic encounter, AI integration may affect the broader professional relationships that support clinical development and practitioner wellbeing. Traditional psychological practice has relied heavily on informal peer consultation. These refer to those “hallway conversations” where psychologists process difficult cases, uncertainties are normalized, clinical wisdom is shared and mutual support is provided. A robust body of research highlights that these kinds of relationships and professional networks helps support clinical decision-making, buffer against the onset of burnout, and help maintain collective standards of care (Norcross and Lambert, 2018). While no empirical studies have directly examined whether AI use reduces informal professional consultation among psychologists, research in healthcare domains suggests this concern warrants attention.

The integration of AI into daily workflows introduces a potential shift in how psychologists seek support (Perivolaris et al., 2024; Smith et al., 2024). Instead of reaching out to a trusted colleague or a mentor when one is uncertain about a diagnosis or treatment plan, it may feel quicker and easier to query a generative LLM (Smith et al., 2024). On the surface, this seems harmless, because efficiency is valued in busy clinical environments (Montani and Striani, 2019). But it risks undermining precisely those relational practices that have historically safeguarded psychologists’ professional judgment. Evidence from digital health and medicine shows that when practitioners rely heavily on technological decision aids, opportunities for peer discussion and collaborative sense-making dramatically decline (Perivolaris et al., 2024; Smith et al., 2024). These reductions are not trivial as collegial exchanges provides critical spaces for stress-testing one’s reasoning, integrating context that algorithms cannot access, and helps to maintain accountability to shared ethical norms (Smith et al., 2024).

The gradual replacement of collegial dialogue or peer consultations with AI recommendations could have cumulative consequences for the profession. First, it may weaken the extent that tacit knowledge is transferred through people. This refers to the subtle judgment calls, clinical instincts, and cultural sensitivities that are rarely written in textbooks but actively shapes effective practice (Perivolaris et al., 2024). Second, when an entire profession turns to the same AI systems for guidance, it risks narrowing the diversity of perspectives within the profession as everyone consults the same homogenized source. Research in medical advice networks shows that diversity is essential for innovation and error correction and that the over-centralization of the sources of advice tends to increase risks of blind spots and groupthink (Mascia et al., 2018). Third, it risks isolating practitioners by normalizing solitary engagement with AI systems to help solve complex problems rather than through collaborative engagement with peers. There is a particular concern of this happening to those working in private practice or who are based in rural contexts. Finally, it may reduce opportunities for mutual support and reflection which is vital to help protect against professional burnout. While direct empirical evidence in psychology is not yet available, these plausible mechanisms, grounded in related fields, suggest that an over-reliance on AI might erode the very networks of human connection that have sustained the profession.

### Training, supervision, and generational transmission of expertise

The integration of AI into psychological training and supervision introduces complex challenges for how professional expertise is developed and carried across generations. Supervisors face difficulty evaluating an intern psychologist’s competence when AI tools are used to complete clinical tasks such as case formulation or treatment planning. When an intern presents an excellent case conceptualization, determining whether this reflects genuine skill development or effective AI use becomes problematic (Van Zyl, 2026a). This ambiguity complicates the objective evaluation of clinical reasoning and risks that interns may advance through training programs without developing the independent clinical reasoning abilities necessary for independent practice. Supervision has traditionally relied not just on evaluating performance in clinical tasks but on observing how and where intern psychologists struggle, where they hesitate, what errors they make in their judgment and how they resolve uncertainty (Rothwell et al., 2021). This process exposes the reasoning behind interns’ clinical decisions and enables supervisors to provide targeted feedback that helps with professional growth. When AI is introduced into these processes, outputs might look professional and polished but obscure the underlying struggles that are essential for learning. Reviews of supervisory practices in healthcare highlight that a trainee’s development is directly dependent upon the quality of feedback received from supervisors, which is usually less about the final product produced and more about their thought processes underpinning it (Rothwell et al., 2021).

The fundamental question of what competencies intern psychologists need in an AI-augmented field remains unresolved (Hutnyan and Gottlieb, 2025). There are currently no clear standards for what constitutes foundational AI knowledge and literacy in psychology nor how these should be trained (Shoemaker et al., 2025). Training programs therefore face difficult decisions about AI skills development and tool integration. Insufficient exposure to AI tools will leave graduates unprepared for contemporary practice where these technologies are increasingly used, but excessive reliance on AI during training may prevent the development of foundational psychological competencies that remain essential despite technological advancement. This creates inconsistency across training programs and could potentially compromise the quality of future practitioners these institutions produce, threatening national and international coherence in what it means to be a competent psychologist. The profession has yet to establish clear guidelines about required competencies, what tools to be used, and when trainees should use AI assistance versus developing skills independently (Hutnyan and Gottlieb, 2025). Failure to calibrate these learning trajectories risks creating cohorts of new psychologists that show exceptional performance when AI is available but brittle, ungrounded expertise when that support is removed or not available.

## Data governance and vendor dependence

At the heart of every AI system lies data. AI integration fundamentally alters how client information is collected, stored, processed, controlled, and potentially used to refine existing models. In traditional practice, psychologists retained direct control over their records, ensuring confidentiality. Contemporary AI platforms rely on cloud-based infrastructure, proprietary algorithms, and layered data-sharing arrangements that shift authority from psychologists to external vendors (see Table 6).

**Table 6:** Data governance and vendor dependence: confidentiality, transparency, and control risks.

Issue/mechanism	Description	Impact on psychological practice
Expanded confidentiality risks	Sensitive client data now passes through several external platforms and vendors, often without full transparency or control.	Increased privacy breaches, loss of client trust, ethics complaints, and liability even when the problem originates elsewhere.
Weak anonymization and cross-border data flows	“De-identified” data can often be traced back to individuals, and storage in other countries exposes it to foreign laws.	Breached consent expectations, privacy violations, and complicated compliance duties across different legal systems.
Transparency gaps and missing audit trail	AI tools may omit, invent, or distort information, leaving no clear record of how an output was created.	Harder to defend professional reasoning, teach good documentation, or ensure valid consent for AI-assisted records.
Overdocumentation and discoverability risk	AI systems record everything—including side comments or preliminary ideas—and retain it indefinitely.	More material becomes legally accessible, increasing practitioner liability and risk of harm if misinterpreted.
Model instability and silent updates	AI systems change constantly as vendors retrain or modify them without notice, shifting how outputs are produced.	Unpredictable behavior, inconsistent accuracy, disrupted routines, and reduced trust in clinical decision support.
Vendor lock-in and loss of autonomy	Closed systems and high switching costs make it difficult to leave or challenge a vendor once embedded.	Less freedom to choose tools, dependence on commercial interests, and workflows that may not serve client needs.

### The multiplication of confidentiality risks

*Confidentiality* is one of the cornerstones of psychological practice, yet AI integration is systematically starting to undermine this foundational building block of the profession. Recent empirical audits of mental health systems show that many apps and AI-driven tools collect extensive amounts of sensitive data without necessary data security measures to ensure privacy (Iwaya et al., 2023). A comprehensive analysis of 27 top-ranked mental health applications revealed alarming inconsistencies in data security, with many failing to use basic safeguards such as encryption or secure authentication to protect client data (Iwaya et al., 2023). The problem deepens when we consider that even data anonymization practices tend to provide less protection than commonly assumed (Murdoch, 2021). Research has shown that machine learning models can easily re-identify the majority of individuals in ostensibly de-identified datasets, exposing both adults and children to renewed privacy risks (Murdoch, 2021). When practitioners enter client data into AI systems, that information may travel through multiple jurisdictions, be stored indefinitely on servers beyond the clinician’s control, accessed by unknown numbers of technical personnel, and processed for purposes extending far beyond the immediate therapeutic encounter (Murdoch, 2021). Each transfer point represents a potential point of compromise. The Federal Trade Commission (FTC), 2023 complaint against BetterHelp for sharing mental health intake data with advertisers highlights how commercial incentives often outweigh privacy protections (Federal Trade Commission (FTC), 2023). When AI systems process clinical notes, audio recordings, or session transcripts, the confidentiality landscape shifts in ways that are difficult to detect and harder to explain during informed consent discussions.

Public awareness of these risks is also increasing, which translates into profession-wide mistrust of AI-related tools. A 2018 survey of 4,000 American adults found that only 11% were willing to share health data with technology companies, compared to 72% with physicians, and only 31% expressed confidence in technology companies’ data security practices (Murdoch, 2021). Despite this well-documented level of mistrust, many hospitals and healthcare organizations continue to share patient data with external services that is not fully anonymized, creating circumstances where psychologists lose control over client information the moment they enter it into AI-augmented systems. The implications extend beyond individual privacy violations and could threaten the profession’s foundational trust (Yadav et al., 2023). Breaches of confidentiality violate ethical codes while simultaneously eroding public confidence in psychological practice. If psychologists rely on systems known to misuse data, they risk reputational harm and weakened therapeutic relationships with their clients (Arvai et al., 2025), even when data breaches occur outside of their direct control (Yadav et al., 2023). Confidentiality risks escalate with every additional vendor and data pipeline involved in AI-assisted workflows, creating a complex web of vulnerability that most practitioners neither fully understand nor can adequately explain to their clients (Yadav et al., 2023).

### Transparency gaps and data-related accountability challenges

Although confidentiality violations represent an immediate and visible threat to psychological practice, a more serious risk lies in how AI systems obscure the very foundations of clinical accountability. When AI tools synthesize information from multiple sources to generate clinical notes, treatment recommendations, or assessment reports, practitioners often cannot identify the specific data sources, reasoning processes, or evidence bases that informed the output (Murdoch, 2021). In legal proceedings such as custody evaluations or malpractice litigation, psychologists may find themselves unable to explain how clinical decisions were reached or fail to demonstrate the factual basis for AI-influenced recommendations they opted to implement. Recent evidence demonstrates the scope of this problem. According to Topaz et al. (2025), ambient AI scribes and note takers frequently produced hallucinated content, omitted clinically relevant details, misattributed statements, and displayed unequal transcription accuracy across different dialects and accents. Because LLMs generate text through statistical predictions and not explicit reasoning, psychologists cannot reliably distinguish which parts of a transcribed record accurately reflect the therapeutic encounter and which were hallucinated by the system (Topaz et al., 2025). If practitioners cannot explain how an AI system produced a conclusion or why certain details were emphasized or excluded, clients cannot meaningfully consent to how their sensitive information is being processed or recorded (Van Zyl, 2026a, b). Psychologists thus face an untenable situation of using tools whose inner workings they cannot account for, while clients retain a right to transparency and informed participation.

The problem extends beyond individual accountability to threaten the profession’s capacity to transmit clinical expertise. Documents generated throughout psychological practice have never served just one purpose. These records note clinical observations and reasons for decisions, but critically, they also externalize clinical reasoning in ways that make that reasoning available for review, supervision, teaching, and quality improvement. When documentation is generated by opaque algorithms or if hallucinations slip into the transcription process, these reflective functions collapse. Supervisors cannot trace how interns reached their clinical conclusions, reviewers cannot trace the basis for interventions, and the iterative refinement of professional judgment is interrupted. There is also a risk that AI systems start to over-document things. Unlike human practitioners who selectively record clinically relevant information, AI systems tend to capture every casual remark, preliminary hypothesis, and speculative interpretation (Lawrence et al., 2025). This exhaustive level of documentation becomes problematic in legal proceedings where every recorded observation can be scrutinized, decontextualized, and potentially misinterpreted (Lawrence et al., 2025). Practitioners may inadvertently create records that harm clients’ interests or expose themselves to professional liability without being aware that problematic documentation is being generated. The inability to control what AI systems choose to document reflects a deeper loss of professional control. Psychologists may no longer exercise judgment about what goes into clinical records, yet they remain accountable for its contents.

### Model instability and the disappearing ground

AI systems differ from traditional clinical tools in one crucial respect: they do not remain stable over time. Unlike traditional online psychometric instruments or therapeutic protocols that maintain consistency across years of use, AI models are dynamic products subject to continuous retraining, updates, and interface changes. Machine learning algorithms are highly sensitive to environmental changes and liable to performance decay even after successful integration into practice (Feng et al., 2022). Practitioners increasingly report that tools they rely on begin generating different types of recommendations, lose previously available features, or alter workflows without prior warning or explanation. This proves particularly problematic when practitioners have developed clinical protocols around specific AI capabilities or trained staff on interfaces that suddenly transform. Many AI vendors provide no version control, update logs, or change documentation, leaving practitioners unaware that the tool they are using may have fundamentally shifted since their last training.

The problem extends beyond planned changes. Dataset drift refers to the tendency of AI performance to degrade when the data it encounters in practice differs from the data on which it was trained (Finlayson et al., 2021). In healthcare, temporal changes in patient populations, diagnostic practices, or information systems mean that the statistical environment is never static, and evidence shows these shifts can cause significant performance deterioration in clinical AI models over time (Davis et al., 2022; Finlayson et al., 2021). For psychologists, this means tools trained on past data may steadily lose accuracy as therapeutic approaches evolve, client demographics change, or new forms of symptom expression emerge. Without ongoing recalibration, AI recommendations risk becoming misaligned with current realities. A once-reliable recommendation system may subtly misclassify, under-detect, or over-pathologize cases, placing clinicians in the untenable position of using tools whose outputs no longer align with current clinical realities (Feng et al., 2022).

For psychologists, this instability creates both immediate and systemic consequences. In the short term, sudden shifts in tool behavior can disrupt established workflows, diminish trust in decision support, and generate errors when recommendations no longer align with practitioners’ expectations. These disruptions introduce ethical stress when practitioners realize that the guidance they are providing may be based on unstable or outdated algorithmic reasoning, reducing their confidence in their own professional judgment, heightening anxiety about potential liability, and reducing willingness to use technological innovations in the future. Over time, reliance on unstable systems corrodes the profession’s ability to validate evidence-based interventions, since the very tools guiding decisions do not remain constant. This unpredictability erodes the basis of evidence-based practice, where treatment rests on the assumption that tools remain stable enough for outcomes to be evaluated, replicated, and compared over time. What emerges is a paradox: the same technologies marketed as increasing reliability and precision actually destabilize the foundations of psychological practice by creating a moving target that practitioners cannot anticipate, undermining professional competence, reducing autonomy, and eroding trust in their own clinical decision-making skills.

### Vendor dependence and lock-in

Another underappreciated challenge lies in the structure of the AI marketplace itself. Much like the history of electronic health record (EHR) systems, AI tools often operate in closed ecosystems that make it difficult or impossible to export data into compatible formats (Adler-Milstein and Pfeifer, 2017). Economic analyses and policy reviews describe how proprietary standards and information blocking practices by venders create switching costs that is so expensive that it entrenches vendors deep within an organizations business ecosystem (Autio et al., 2023; Jeyaseelan, 2025). These practices generate high switching costs that effectively lock psychologists and organizations into long-term vendor relationships even when the evidence shows that these tools cause harm or are ineffective (Jeyaseelan, 2025). These dependencies extend beyond data portability to include workflow integrations, staff training investments, and billing arrangements which all together make transitions very expensive and disruptive (Jeyaseelan, 2025).

The consequences of this vendor lock-in are rather significant (Jeyaseelan, 2025). When these venders change their pricing models, discontinue certain services, or alter platform features, practitioners are faced with a lose–lose decision (Autio et al., 2023). They either accept new unfavourable terms or have to undergo costly transitions that may involve data loss and clinical downtime (Jeyaseelan, 2025). The concentration of AI development among a handful of powerful technology companies may intensify this imbalance (Topol, 2019). By shaping the features and limitations of tools, these vendors indirectly dictate how psychological practice is conducted, documented, and evaluated (Topol, 2019). Their commercial priorities (like maximizing engagement metrics or expanding data collection for secondary use) often eclipse psychologist’s professional values which forces them to work within frameworks that were designed to serve corporate- rather than clinical interests (Topol, 2019).

For individual practitioners and small practices, this level of asymmetry power is even starker. Few independent psychologists have the leverage to negotiate their contractual terms, or request features that are aligned with the therapeutic needs of their clients (Murdoch, 2021). And almost none have the power to ensure that the systems they are locked into will always reflect their professional ethical values (Topol, 2019). Instead, they find themselves becoming dependent on tools that’s built by vendors with little understanding of, or accountability to, the practice of psychology. This represents a structural transfer of authority where professional judgment and autonomy are gradually ceded to vendors whose goals are driven by profit rather than client care (Jeyaseelan, 2025). The result is a profession increasingly shaped by external commercial forces, with psychologists compelled to adapt to tools that neither reflect nor prioritize their core values.

## Physical health and wellbeing

While AI promises to enhance clinical efficacy and reduce administrative burden in psychological practice, emerging evidence suggests it may substantially impact psychologists’ own physical health and wellbeing. The irony is stark: technologies designed to alleviate professional stress may instead create new forms of psychological strain and physical health problems that threaten practitioner sustainability. Understanding how AI use affects psychologists’ physical and psychological functioning is essential for developing protective strategies that allow practitioners to harness AI benefits while preserving their health. Below we discuss the main ways AI use can impact wellbeing (c.f. Table 7).

**Table 7:** Physical health and wellbeing: technostress, workload, and somatic costs.

Issue/mechanism	Description	Impact on psychological practice
Digital- and techno stress	Constantly learning, monitoring, and adapting to new AI tools creates overload, blurs work–life boundaries, and fuels job insecurity.	Heightened stress and burnout, reduced emotional presence with clients, more mistakes under pressure, and stronger intentions to leave the field.
Rising job demands despite automation	Instead of saving time, AI often adds new responsibilities like checking outputs, fixing errors, and managing interfaces.	Longer hours, less reflection and peer contact, rising workload, and declining job satisfaction.
Economic strain of adoption	Ongoing software, training, and equipment costs strain finances, especially for solo or small-practice psychologists.	Increased financial stress, higher caseloads or shorter sessions, and widening inequality between well- and under-resourced practitioners.
Physical strain and sleep disruption	Extended screen time, static posture, and late-night digital work harm physical health and sleep patterns.	Fatigue, loss of focus and empathy, slower recovery, and cumulative health risks over time.
Algorithmic dependence	Continuous reliance on AI erodes confidence in personal judgment and can create emotional or cognitive attachment to the system.	Hesitation to make independent decisions, weaker critical thinking, distress when tools fail, and subtle identity confusion.
AI psychosis-like phenomena	Deep immersion with humanlike AI can blur reality boundaries and reinforce distorted thinking in stressed or vulnerable users.	Impaired reality testing, risky judgment calls, emotional exhaustion, and the need for rest or supervision to restore balance.

### Digital stress and technostress in psychological practice

*AI-related technostress* is emerging as one of the predominant occupational health concerns among psychologists navigating the demands of constantly evolving AI technologies. This distinctive form of psychological strain arises from the emotional, cognitive, and professional demands that AI technologies place on practitioners’ time, attention, and clinical skills (Lițan, 2025a). The cumulative effect of these techno-stressors creates chronic stress responses that progressively deplete psychologists’ emotional and cognitive resources, leading to documented increases in psychological distress, common mental health problems, negative affect, and even burnout (Issa et al., 2024; Lițan, 2025a).

According to Chang et al. (2024) and Issa et al. (2024), AI-related technostress manifests through six interconnected factors that systematically undermine practitioner wellbeing:

- Techno-overload (i.e., feeling pressured to work faster and harder).
- Techno-invasion (i.e., AI technologies blurring work-life boundaries due to an inability to disconnect from these technologies).
- Techno-complexity (i.e., struggling to understand, or adapt to rapidly evolving AI systems).
- Techno-insecurity (i.e., fear of being replaced by AI technologies).
- Techno-uncertainty (i.e., anxiety about constant changes in AI technologies).
- Techno-unpredictability (i.e., stress associated with the unpredictability of the outcomes associated with and outputs of AI technology systems).

Research in healthcare settings reveals how these Ai-related techno-stressors emerge in practice. Over-burdened practitioners must constantly learn and monitor new AI systems while verifying outputs and managing system updates with very limited guidance (Chang et al., 2024). This increased level of cognitive load or the cognitive burden of continuous adaptation creates what Ragolane and Patel (2025) term “AI (change) fatigue” (i.e., a state of exhaustion stemming from continuous AI driven technological adaptation that diminishes both professional satisfaction and clinical effectiveness). The psychological toll extends beyond immediate stress responses to fundamentally threaten the professional resources (i.e., autonomy, competence, and job security) that psychologists value (Lițan, 2025b). When these core resources are constantly undermined, practitioners experience the emotional exhaustion and depersonalization characteristics that associated with severe burnout (Kaltenegger, 2024).

The impact on wellbeing manifests across multiple dimensions of professional life. Studies demonstrate that technostress and change fatigue not only precipitate stress, depression, anxiety, and burnout but also drive intentions to leave organizations or abandon the profession in its entirely (Issa et al., 2024; Kaltenegger, 2024). Prolonged exposure to techno stress also translates into organizational withdrawal behaviors at the physical- (e.g., absenteeism, quite-quitting), psychological- (e.g., reduced job involvement) and social levels (e.g., poor pro-social behaviors) (c.f. Bondanini et al., 2020). For psychologists, technostress represents more than just a technological frustration as it fundamentally alters the nature of their therapeutic work and professional identity. The burden is especially challenging because practitioners must simultaneously manage their own technological adaptation while also needing to maintain therapeutic presence with their clients. This dual demand places an extra layer of stressors on a psychologist that taxes their already limited emotional and cognitive resources. The constant connectivity demanded by AI systems also creates “techno-invasion,” where boundaries between work and personal life dissolve as practitioners feel compelled to engage with AI tools outside traditional work hours for documentation, training, or system management. This constant connectivity reduces the restorative time needed for preventing burnout and maintaining psychological wellbeing.

Research further reveals a particularly concerning cycle affecting practitioner wellbeing. Mental health professionals with more direct interaction with digital technologies tend to report higher stress levels and lower digital competence, creating a self-perpetuating pattern where stress impedes learning, which generates additional stress (Golz et al., 2021). This cycle is particularly problematic given that effective psychological practice requires emotional availability and cognitive flexibility which are both resources that technostress systematically depletes (Golz et al., 2021). The impact appears particularly acute among older practitioners and may impact their decision to retire early or reduce their active involvement at work (Golz et al., 2021). This may reduce workforce diversity and remove experienced clinical wisdom from practice in a time where this is drastically needed to guide ethical AI integration.

Taken together, the cumulative effect of AI-related technostress on psychologist wellbeing might not just negatively affect the individual practitioner but may threaten the sustainability of the mental health profession itself. As practitioners struggle with adapting to new technological demands while also attempting to maintain therapeutic effectiveness, the profession faces a crisis where the tools intended to enhance practice may instead be driving talented clinicians away from the field, they once found meaningful and fulfilling.

### Paradoxical increases in job demands and economic strain

The integration of AI into psychological practice may also paradoxically increase (rather than decrease) a psychologist’s job demands. Rather than alleviating work-pressure, AI technologies often creates additional tasks, places new cognitive burdens, and acts as a new source of uncertainty that extend beyond traditional clinical roles.

The first major increase in job demands is experiences of job insecurity due to AI systems automating core aspects of a psychologist’s job (e.g., assessment, diagnosis, and even elements of the therapeutic process). This growing anxiety (referred to earlier as techno-insecurity) is caused by a persistent fear of being replaced by technology or that one will be outperformed by colleagues with better AI related technical expertise (Chang et al., 2024). However, studies show that these concerns are not evenly distributed among all population subgroups. Younger professionals and women report higher levels of techno-insecurity which is often linked to worries about career advancement or professional marginalization within increasingly digital workplaces (Chang et al., 2024; Rašticová et al., 2025). The job demands-resources model suggests that when job demands increase without corresponding increases in resources or control, the resulting imbalance creates conditions for burnout and professional exhaustion (Bakker and Demerouti, 2024).

Workload also expands with AI integration as psychologists must now manage both traditional clinical responsibilities and the new demands mastering new AI technologies brings (Olson et al., 2025). While AI promises efficiency gains, research reveals that practitioners spend considerable time learning new systems, troubleshooting technical problems, correcting AI errors, and managing the increased documentation that AI systems generate (Albrecht et al., 2025; Olson et al., 2025). The concept we discussed earlier about digital note-takers and “over-documentation” illustrates this paradox clearly. AI systems’ capacity to capture and process detailed data, creates more documentation that a psychologist needs to review, edit, and manage (Albrecht et al., 2024). This changes what was intended to be a time-saving technology into a source of additional work (Albrecht et al., 2024). Olson et al. (2025) showed that professionals allocate almost half their workday to documentation and administrative tasks even with AI assistance. This suggests that these technologies may be redistributing workload to other tasks rather than reducing it.

Finally, the complexity of an AI-augmented practice also creates new forms of cognitive and emotional labour that traditional training did not prepare psychologists to manage. Practitioners must now serve as technology troubleshooters, data quality monitors, and act as supervisors for algorithms while also having to maintain their primary roles as therapists. In this hybrid role, they must now manage both human complexity and machine uncertainty. Emerging research in digital labour suggests that when AI handles routine or low-complexity tasks, professionals are left with a narrower portfolio of high-stakes, emotionally intense cases by removing the level of job variety that once mitigated stress (Huo et al., 2025; Pavuluri et al., 2024). When all one’s work is concentrated in more challenging, high-stakes domains, it increases the complexity of a psychologist’s work which also increases their job-related stress and reduces job dissatisfaction (Pavuluri et al., 2024). Because most health systems provide no additional time, training, or institutional support for managing these new demands, these cumulative pressures can degrade practitioner wellbeing and, ultimately, negatively affect the quality of client care.

### Economic pressures and stress

The financial burden of AI adoption creates substantial economic stress for psychologists, with those in private practice and under-resourced areas potentially suffering the most. While there are a significant amount of benefits stemming from AI technologies, the economic reality is that it costs significant upfront investments and requires ongoing operational costs that many practitioners struggle to afford. Comprehensive AI systems require not only software subscriptions but also hardware upgrades, system integration expenses, training costs, and technical support fees that collectively represent thousands of dollars in annual expenditure. These financial demands occur within a healthcare system that rarely acknowledges or compensates for technology-related practice expenses. This forces practitioners to absorb these costs which directly impacts the sustainability of their practice and their personal, financial wellbeing.

But it also creates a two-tier system where well-resourced practices can afford sophisticated AI tools while practitioners serving lower-income communities face difficult choices between financial sustainability and technological competitiveness. Psychologists serving lower-income or marginalized communities are especially vulnerable because their revenue streams may already be constrained and their access to capital limited. In these contexts, the decision to adopt AI becomes a high-stakes gamble: either invest heavily and hope for return, or risk falling behind but preserve financial stability. This dynamic can exacerbate inequalities in care quality and clinician wellbeing across settings. Some technology adoption frameworks warn that cost barriers amplify disparities when the very practices most in need of innovation can least afford it (Berardi et al., 2024).

The economic strain of AI adoption could also trigger compensatory behaviors that could further compromise psychologists’ wellbeing. Interviews with mental health providers confirm that cost is a recurring barrier where many report that potential benefits of AI are overshadowed by uncertainty about sustainability and amortizing costs over time (Zhang and Wang, 2024). To manage these costs, practitioners may resort to shortening session times, increasing client caseloads, or reducing non-billable activities like consultation and professional development. All of these changes contributes to a psychologist’s workload, stress, and reduce the ability to recover between sessions. The economic pressures tied to AI adoption therefore could create a self-perpetuating cycle in which financial strain drives work intensification, erodes rest and reflection, and culminates in burnout which at the same time reduces the quality of care they provide.

### Physical health consequences of extended AI use

While there is extensive literature on computer-related musculoskeletal disorders, digital eye strain, and sleep disruption in general office workers and healthcare populations, the specific impact of AI use on physical health of psychologists remains completely unexplored. This absence is striking given that related research domains provide strong evidence that extended AI and technology use has a direct negative effect on the physical health professional- and medical workers.

The most common physical health symptoms stem from prolonged screen time (Chapala et al., 2025). Research indicates that mental health professionals spend substantial portions of their workday engaged with digital technologies. Studies show that healthcare professionals typically spend 4.5–5.9 h daily using electronic health records and computer-based systems (Arndt et al., 2017), while 38% of surveyed doctors report over 5 h of daily screen time (Chapala et al., 2025). Waersted et al. (2010) found that prolonged screen time (>2 h daily) could lead to significant higher prevalence levels of musculoskeletal disorders affecting the neck, shoulders, and upper extremities. Psychologists using AI-assisted tools frequently toggle between multiple screens (e.g., clinical notes, dashboards, dashboards, and chat interfaces) which create micro-repetitive movements that cause strain and results in sustained static postures that accelerate muscular fatigue. The problem is exasperated by the absence of natural movement that was once embedded in a psychologist’s pre-digital routine (i.e., walking between offices, writing by hand, or accessing physical files). Studies in occupational physiology show that replacing intermittent movement with continuous sedentary behavior disrupts blood circulation, reduces metabolic rate, and elevates inflammatory bio-markers over time (Pinto et al., 2023). These mechanisms provide a strong theoretical foundation for anticipating increased rates of chronic pain, fatigue, and metabolic dysfunction among psychologists whose work now unfolds primarily through digital mediation.

Sleep disturbance represents another major pathway that could like AI over use to impaired physical health. Psychologists often complete their administrative work after clinical consultation hours (Northrup, 2022) which extends their exposure to blue light and cognitive stimulation late into the evening (Zhong et al., 2025). Empirical evidence shows that evening screen exposure suppresses melatonin production and disrupts one’s circadian rhythms, while the cognitive stimulation delays the onset of sleep and reduces one’s overall sleep quality (Zhong et al., 2025). Studies document that mental health professionals using AI-assisted documentation systems report working later into the evening to review and correct AI-generated content (Olson et al., 2025) which could create a pattern of delayed sleep phase and chronic sleep deprivation (Zhong et al., 2025). The physiological consequences of disrupted sleep impairs immune functioning, increases risk of inflammation and increases one’s risk for cardiovascular disease. These negative health implications may accumulate over years of practice and create significant morbidity. For the psychologist, chronic sleep restriction also undermines their ability to focus, regulate attention, emotional attunement and empathic accuracy (Northrup, 2022). AI technologies can thus indirectly contribute to somatic risk not just through physical mechanisms alone, but by altering temporal boundaries between work and rest in ways that the body cannot easily compensate for.

A subtler but equally concerning mechanism involves the physical activity paradox. AI technologies where designed to help reduce the cognitive, emotional and physical effort we exert but they may also inadvertently amplify sedentarism. In principle, AI tools aim to make practice more efficient but in reality, they often tether psychologists to their desks for longer periods as they review and refine outputs, respond to digital prompts, or navigate updated interfaces. The result in what we call “digital immobility” which describes the extended screen-based work that suppresses spontaneous movement and promotes physiological stagnation. Studies among healthcare professionals demonstrate that this kind of immobility correlates with elevated C-reactive proteins, increased blood pressure, and greater self-reported fatigue (Kaltenegger et al., 2023). This implies that extended AI use my physically anchor practitioners in positions that compromise their vascular health and metabolic balance (Kaltenegger et al., 2023).

In sum, while the literature is still to investigate the physical health consequences of AI use among psychologists, converging evidence from occupational medicine and digital health shows that there is a significant among of risk involved. Sustained screen exposure, reduced movement, disrupted sleep, and physiological stress responses form a constellation of interrelated vulnerabilities that may gradually affect practitioners’ health over time. Without proactive strategies to mitigate ergonomic strain, reestablish boundaries around rest, and reintroduce movement into digital workflows, the physical wellbeing of psychologists’ risks becoming another unintended casualty of AI-driven efficiency.

### Psychological dependency on AI systems

While psychological dependencies on AI and AI-induced psychotic phenomena are primarily documented in client populations, the intensive professional use of AI systems may create similar vulnerabilities in psychologists themselves. *Algorithmic dependence* refers to an over-reliance on AI for core steps of case conceptualization, treatment planning, and psychoeducation where clinical conclusions are drawn primarily from AI systems rather than from foundational theoretical knowledge/experience (Budzyń et al., 2025). For psychologists, this may manifest as excessive trust in algorithmic recommendations, reluctance to make independent decisions, or anxiety when required to work without digital assistance. The concept of “epistemic dependence” describes how practitioners may develop excessive reliance on AI systems for clinical reasoning and decision-making which results in a form of cognitive dependency on these systems where independent professional judgment tends to deteriorate over time (Boyd and Markowitz, 2025). This dependency does not develop out of a practical reliance on the technology but rather as a result of a psychological attachment to AI systems’ perceived objectivity and authority (Naseer et al., 2025). The boundary between tool use and psychological dependence blurs when practitioners begin viewing AI as essential to their professional competence rather than as supplementary support (Naseer et al., 2025).

This level of dependency may develop out of the anthropomorphiscation of AI systems (Wan and Chen, 2021). This occurs when practitioners start to attribute human-like qualities like competence, empathy, or authority to these in-human AI systems when these capacities do not really exist (Li and Suh, 2022). Studies show that anthropomorphic design features enhance users’ trust and emotional connection to AI systems which facilitates deeper psychological dependencies that can compromise one’s professional objectivity (Wan and Chen, 2021). This tendency is amplified in professional contexts where AI systems appear to “understand” clinical presentations or offer sophisticated diagnostic insights that seem clinically insightful (Li and Suh, 2022). Because these systems simulate empathy and comprehension, practitioners may unconsciously treat them as quasi-colleagues and start forming the same patterns of emotional reliance they would during human collaboration (Longoni et al., 2019). These kinds of affective dynamics reinforce the psychological attachment to the machine’s validation (Longoni et al., 2019).

This anthropomorphization becomes particularly impactful in therapeutic contexts, where relational attunement and professional validation are central to a psychologist’s identity (Longoni et al., 2019). The emotional reassurance psychologists experience when AI systems confirm their assessments can therefore create a powerful reinforcement loop. Practitioners begin to trust AI validation over their own judgment and experiencing unease or self-doubt when the system disagrees with them. This could eventually lead to practitioners gradually preferring the AI’s consistent and reassuring recommendations over the unpredictability of human consultation (Longoni et al., 2019). Research in the medical sciences demonstrates this clearly. Medical practitioners report positive emotional reactions (e.g., feeling reassured) to AI systems that confirm their assessments and negative emotional reactions (e.g., discomfort or anxiety) when AI outputs diverge from their original conclusions (Longoni et al., 2019). Over time, the boundary between collaboration and psychological dependence starts to blur as what began as instrumental reliance on a tool, could evolve into an emotional and epistemic bond that subtly reshapes self-efficacy and professional identity (Li and Suh, 2022; Longoni et al., 2019).

The practical consequence of this kind of psychological dependency may manifest in a number of ways. Overreliance on AIs can negatively affect one’s capacity for critical thinking, professional autonomy and create a form of cognitive dependency that resembles technological addiction more than informed tool use. This dependency becomes particularly problematic when AI systems are offline, when a client presents with a novel set of symptoms that require creative problem-solving, or when clients present with complex, multifaceted difficulties that resist simplification into the categories that AI systems can recognize (Budzyń et al., 2025). In a sample of medical professionals, this level of dependency was so deeply ingrained that respondents reported feeling anxious, emotionally stressed as well as experienced decision paralysis when they were required to work without AI assistance (Naseer et al., 2025).

Beyond these practical implications, intensive AI use may also contribute to more subtle changes in psychologists’ reality testing abilities. Extended engagement with AI systems that simulate therapeutic dialogue or that provided sound clinical reasoning may create confusion about the boundaries between human and artificial intelligence (Lee and Lee, 2023). Some practitioners report experiencing uncanny valley effects (i.e., the psychological discomfort from AI systems that seem almost but not quite human) that create persistent feelings of unease when these tools are actively used in their professional practice (Lee and Lee, 2023). Research in human-computer interaction suggests that this anthropomorphization of AI systems, even in professional contexts, can lead to inappropriate emotional investments and distorted perceptions of AI capabilities that could compromise their professional judgment.

A related phenomenon involves the emergence of parasocial relationships with AI tools. Parasocial relationships are one-sided emotional attachments to media, public figures or even AI systems in which one feels a level of intimacy or connection despite the fact that there is no reciprocal interaction (Morrin et al., 2025). In AI contexts, users sometimes name their LLM chatbots thereby imbuing them with a level of personal identity and relational meaning which helps them establish deeper parasocial bonds with these AI tools (Maeda and Quan-Haase, 2024). This is particularly problematic when practitioners start to substitute AI recommendations for collegial support or professional development opportunities. Research indicates that users who form strong emotional attachments to AI systems experience grief-like responses when systems update or change, a phenomenon that could disrupt professional functioning if practitioners become overly invested in specific AI tools (drawing on studies of “digital ambiguous loss” documented in client populations) (De Freitas et al., 2024b).

The risk of developing more severe psychological disturbances due to AI use in professionals remains largely theoretical but warrants careful consideration. AI psychosis is an emergent, non-clinical phenomenon where individuals that are intensely interacting with AI systems begin to experience delusion-like thought patterns, impaired reality testing, or blurred boundaries between AI output and personal belief (Østergaard, 2023). The term has gained attention in psychiatric and AI ethics discourse as a way to describe cases in which a user attributes agency, intentionality, or hidden meaning to AI responses or comes to believe that the AI is speaking to them in cryptic or directive ways (Østergaard, 2023). Some conceptual papers warn that AI’s tendency to hallucinate (i.e., produce confident but false statements) and its sycophantic response style (i.e., agreeing with user statements) may reinforce delusional beliefs in vulnerable individuals (Østergaard, 2023).

While case reports of AI-induced psychotic symptoms are generally centred around those individuals who intensively use AI for therapy/companionship, it is plausible that psychologists themselves could become susceptible to AI psychosis. This is because the underlying mechanisms driving AI-induced psychosis (i.e., overexposure, emotional projection onto AI systems, and cognitive dissonance between human and machine reasoning) could theoretically affect practitioners in a similar way when they are under extreme stress or those with a pre-existing vulnerability or mental health condition. As psychologists increasingly rely on AI for reasoning, dialogue scaffolding, or validation of their ideas, the very familiarity and immersion might trigger these symptoms. When a psychologist begins to trust AI outputs as quasi-authoritative or start to attribute nuance or intention within an AI model response, the same dynamics of delusion reinforcement, confirmation bias, and belief entrenchment might apply.

While there is no current reported case of full-threshold AI-induced psychopathology among professionals, the subclinical symptoms including derealization (feeling that one’s professional work is unreal), depersonalization (feeling disconnected from one’s professional identity), and existential confusion about the nature of therapeutic relationships in an AI-mediated world are increasingly being reported. These psychological impacts suggest that the mental health costs of AI integration extend beyond stress and burnout to potentially include alterations in psychological functioning that can compromise both one’s personal wellbeing and professional effectiveness. The absence of systematic research on these phenomena in professional populations represents a critical gap in our knowledge base.

## Discussion

This commentary reveals that AI integration in psychological practice operates through a multilevel cascading system where risks at cognitive, ethical, relational, and physical levels amplify one another rather than functioning as isolated concerns. Four core findings emerge from this discussion:

1. First, cognitive deskilling manifests not merely as reduced diagnostic skill but as a fundamental transformation in how psychologists think, moving from active generators of clinical insight to passive curators of AI-generated content. The evidence shows diagnostic reasoning atrophy through reduced practice of differential diagnosis, decreased confidence in clinical intuition, metacognitive deterioration, and automation bias that persists even among experienced practitioners under time pressure.
2. Second, professional identity erosion occurs through multiple interconnected pathways: fear of replacement creates existential uncertainty about professional value, role fragmentation reduces integrated clinical work to disconnected optimizable subtasks, AI-specific impostor syndrome undermines the link between effort and achievement, and meaning crises emerge at individual, organizational, professional, disciplinary, and societal levels simultaneously.
3. Third, ethical compromise operates through mechanisms beyond individual moral failing, including AI-mediated moral injury where practitioners experience distress from institutional pressures to follow algorithmic recommendations against their professional judgment, moral deskilling where capacity for independent ethical reasoning atrophies through delegation to AI systems, and algorithmic value imposition where developer priorities silently constrain therapeutic pluralism and cultural competence without practitioner awareness.
4. Fourth, these cognitive, identity, and ethical vulnerabilities create conditions for physical and psychological harm including validated measures of technostress that correlate with burnout and intentions to leave the profession, paradoxical increases in workload despite automation promises, economic strain creating two-tier systems, physical health costs from extended screen time and sedentarism, and psychological dependencies where practitioners develop epistemic reliance and emotional attachments to AI systems that compromise professional autonomy.

What distinguishes these findings is not that each represents a novel risk but that together they reveal a recursion problem: psychologists are adopting technologies that fundamentally alter their cognition, emotion, and professional identity without applying the empirical scrutiny they would demand in any other domain of practice.

The implications span practice, education, research, professional governance, and societal responsibility. For practice, the concept of reflective technological integration becomes essential: the deliberate, evidence-based, and ethically grounded use of AI systems in ways that reinforce rather than replace professional judgment. This requires that every technological intervention begins with the question “What human capacities does this preserve, and what does it risk eroding?” *Practitioners* must develop metacognitive awareness of how tools shape their thinking, maintain critical distance from algorithmic recommendations even under time pressure, and cultivate practices that preserve therapeutic presence and unmeasurable capacities like moral discernment and cultural attunement.

For *education and training*, programs face the challenge of preparing psychologists who can work effectively with AI while developing foundational competencies that remain essential when technological support is unavailable. This demands curriculum reforms that include not only AI literacy but critical consciousness about how tools shape cognition, explicit teaching of when to override algorithmic recommendations, supervised practice in working without AI assistance to prevent brittle expertise, and competency standards that ensure graduates can function independently. Training must also address the intergenerational tensions we identified, establishing clear guidelines about when trainees should use AI assistance versus developing skills autonomously.

For *research*, urgent longitudinal and phenomenological studies are needed to clarify how sustained AI engagement alters cognition, emotional regulation, and professional identity over time. Priority questions include: At what point does convenience become dependency? How do practitioners develop and maintain appropriate scepticism toward algorithmic recommendations? What protective factors buffer against technostress and moral injury? How do economic pressures interact with adoption decisions to create inequities? What are the long-term physical health consequences of AI-augmented practice? Research must also establish empirically grounded risk frameworks that move beyond theoretical speculation to document actual harms in real-world practice contexts.

For *professional bodies*, ethical codes and credentialing standards require substantial updates to address algorithmic reliance, technostress management, digital moral injury, vendor dependencies, and preservation of unmeasurable therapeutic capacities. Organizations like the APA and EFPA must provide definitive guidance rather than deferring to vendor-determined standards, establish clear boundaries around appropriate AI use in assessment, diagnosis, and treatment planning, create accountability structures for monitoring psychological impacts on practitioners, and defend the profession’s epistemological authority against encroachment from technology companies and adjacent disciplines.

For *society*, psychology must articulate and defend its distinctive role in honouring irreducible human complexity against reductionist optimization pressures. If the profession cannot maintain practices that recognize suffering as fundamentally human experience demanding empathy and shared meaning-making rather than information processing requiring computational solutions, it risks participating in the cultural impoverishment it should be resisting. The broader implication is that psychology serves as society’s most sophisticated defender of human complexity, and its surrender to algorithmic reduction represents not merely professional transformation but societal loss.

This commentary has several important limitations which may lead to potential suggestions for future research. As a narrative commentary rather than systematic review, we acknowledge potential selection bias in the literature reviewed and absence of formal methodological protocols for evidence synthesis. While we attempted comprehensive coverage, the rapidly evolving nature of AI technologies means some observations may become outdated quickly, and we cannot account for developments occurring after our literature search. The empirical base for some claims, particularly regarding psychologists specifically, remains limited. Much of our evidence necessarily draws from adjacent healthcare and education domains where research is more developed, with the assumption that mechanisms identified in medical practice, nursing, or higher education will operate similarly in psychological contexts. This assumption requires empirical validation. Many observations about long-term effects particularly cognitive deskilling trajectories, identity erosion patterns, and development of psychological dependencies remain speculative, extrapolating from short-term studies or cross-sectional data to propose longitudinal processes that require prospective investigation. This commentary may have over emphasized risks over AI’s benefits in service of highlighting underexplored concerns, though AI offers genuine value when thoughtfully integrated into practice. The purpose was not to reject AI wholesale but to ensure risks receive commensurate attention to benefits in professional discourse. Geographic and cultural limitations exist in the evidence base, with most research conducted in Western, industrialized contexts. How AI impacts professional identity, ethical reasoning, or therapeutic relationships may differ substantially across cultural contexts with different healthcare systems, professional training models, or cultural attitudes toward technology.

The commentary also reflects a particular moment in AI development with the specific focus on current LLM-based systems and ambient AI tools, but future technological developments may introduce novel risks or mitigate identified concerns. Finally, I recognize that individual practitioners vary considerably in their vulnerability to the risks identified, with factors like personality, training background, practice setting, client population, and organizational context all likely moderating effects. This commentary describes general patterns and mechanisms without adequately accounting for this heterogeneity. Despite these limitations, the convergence of evidence across domains and the theoretical coherence of the cascading risk framework suggest these concerns warrant serious professional attention and systematic empirical investigation.

## Conclusion

While AI offers substantial benefits to optimise the efficacy of psychologists, this commentary shows that it might pose significant threats to practitioner competence, client safety, and the integrity of therapeutic relationships. The evidence presents a sobering picture of systematic risks that are emerging faster than our ability to understand or mitigate them. It’s evident that AI should not be seen as a neutral instrument but rather as a co-actor in psychological practice that reshapes how psychologists think, feel, decide, and relate to their work. Drawing learnings from adjacent domains, we can see that AI use has the potential to erode our critical thinking abilities, to distort our level of ethical awareness, and could foster a level of dependency that challenges our understanding of reality. What may being as an effort to enhance our clinical precision or professional effectiveness may, if left unexamined, hollow out the very cognitive and moral foundations on which our discipline is built.

These patterns highlight a deep mismatch between the speed of technological adoption and the pace of our psychological adaptation. Psychologists are entering AI-mediated environments without sufficient conceptual, ethical, or self-regulatory frameworks to manage the psychological effects of their own technology use. This imbalance highlights the urgent need for academic research and professional attention to the matter. Longitudinal and phenomenological research is needed to clarify how sustained AI engagement alters cognition, emotional regulation, and professional identity over time. Equally urgent is the need for practitioner education. Psychologists must be equipped with the tools to not only ethically and responsibly use AI, but also to recognize their potential for negatively affecting their professional functioning and wellbeing. Creating awareness and developing AI literacy constitute the first line of defence against the onset of these potential negative consequences.

In order to safeguard the future of the profession, there should be a collective commitment to what can only be described as *reflective technological integration*: the deliberate, evidence-based and ethical use of AI systems in ways that reinforces rather than replaces a psychologist’s professional judgment. Professional bodies must update their ethical codes and training standards to include guidance on algorithmic reliance, technostress management, and digital moral injury. Researchers must establish empirically grounded risk frameworks, while organizations should implement structures that monitor the psychological impacts of AI use staff. Ultimately, the measure of progress will not be the sophistication of the AI systems we use but rather related to the extent that we can preserve (and augment) the human capacities that define psychology at its best. The profession stands at a crossroads: to remain human-centred in an algorithmic age, psychologists must become as fluent in self-reflection about their tools as they are skilled in their clinical use. But the window for proactive intervention is rapidly closing, so the time for action is now.
